# Compounding *Achromobacter* Phages for Therapeutic Applications

**DOI:** 10.3390/v15081665

**Published:** 2023-07-30

**Authors:** Ana Georgina Cobián Güemes, Tram Le, Maria Isabel Rojas, Nicole E. Jacobson, Helena Villela, Katelyn McNair, Shr-Hau Hung, Lili Han, Lance Boling, Jessica Claire Octavio, Lorena Dominguez, Vito Adrian Cantú, Sinéad Archdeacon, Alejandro A. Vega, Michelle A. An, Hamza Hajama, Gregory Burkeen, Robert A. Edwards, Douglas J. Conrad, Forest Rohwer, Anca M. Segall

**Affiliations:** 1 Department of Biology, Viral Information Institute, San Diego State University, San Diego, CA 92182, USA; 2 Marine Microbiomes Lab, Red Sea Research Center, King Abdullah University of Science and Technology, Building 2, Level 3, Room 3216 WS03, Thuwal 23955-6900, Saudi Arabia; 3 Computational Sciences Research Center, San Diego State University, San Diego, CA 92182, USA; 4 Research Centre for Eco-Environmental Sciences, Chinese Academy of Sciences, Beijing 100085, China; 5 College of Biological Sciences, University of California Davis, Davis, CA 95616, USA; 6 David Geffen School of Medicine, University of California Los Angeles, Los Angeles, CA 90025, USA; 7 Flinders Accelerator for Microbiome Exploration, Flinders University, Sturt Road, Bedford Park 5042, Australia; 8 Department of Medicine, Division of Pulmonary, Critical Care and Sleep Medicine, University of California San Diego, San Diego, CA 9500, USA

**Keywords:** *Achromobacter* phage, phage therapy, prophage induction, phage production

## Abstract

*Achromobacter* species colonization of Cystic Fibrosis respiratory airways is an increasing concern. Two adult patients with Cystic Fibrosis colonized by *Achromobacter xylosoxidans* CF418 or *Achromobacter ruhlandii* CF116 experienced fatal exacerbations. *Achromobacter* spp. are naturally resistant to several antibiotics. Therefore, phages could be valuable as therapeutics for the control of *Achromobacter*. In this study, thirteen lytic phages were isolated and characterized at the morphological and genomic levels for potential future use in phage therapy. They are presented here as the *Achromobacter* Kumeyaay phage collection. Six distinct *Achromobacter* phage genome clusters were identified based on a comprehensive phylogenetic analysis of the Kumeyaay collection as well as the publicly available *Achromobacter* phages. The infectivity of all phages in the Kumeyaay collection was tested in 23 *Achromobacter* clinical isolates; 78% of these isolates were lysed by at least one phage. A cryptic prophage was induced in *Achromobacter xylosoxidans* CF418 when infected with some of the lytic phages. This prophage genome was characterized and is presented as *Achromobacter* phage CF418-P1. Prophage induction during lytic phage preparation for therapy interventions require further exploration. Large-scale production of phages and removal of endotoxins using an octanol-based procedure resulted in a phage concentrate of 1 × 10^9^ plaque-forming units per milliliter with an endotoxin concentration of 65 endotoxin units per milliliter, which is below the Food and Drugs Administration recommended maximum threshold for human administration. This study provides a comprehensive framework for the isolation, bioinformatic characterization, and safe production of phages to kill *Achromobacter* spp. in order to potentially manage Cystic Fibrosis (CF) pulmonary infections.

## 1. Introduction

*Achromobacter* spp. were identified as the dominant member of the microbial community in sputum samples from two patients with cystic fibrosis (CF) who suffered acute and fatal exacerbations. *Achromobacter* spp. are Proteobacteria of the order Burkholderiales; they can use anaerobic metabolism in the presence of nitrate or nitrite, and can use denitrification for respiration. *Achromobacter* spp. are long-term colonizers of CF lungs [1], and are of increasing concern. *Achromobacter xylosoxidans* isolated from CF patients has pathogenic characteristics such as the presence of toxins and other virulence factors [2]. In addition to infections in CF lungs, *Achromobacter* spp. have been reported to cause urinary tract infections [3], endocarditis [4], meningitis [5], and ocular infections [6].

Bacteriophages (phages) have been used as therapeutics to modify the microbiome and aid in the clearance of bacterial infections [7,8]. Phage therapy (PT) has been used to treat microbial infections in the respiratory airways of CF patients such as *Mycobacterium abscessus* [9], *Pseudomonas aeruginosa* [10], and *Achromobacter* spp. [11]. The use of phages as therapeutics has been empirically-based, and the phages applied to patients are not always characterized [12]. Genomic characterization of phages to screen for lysogenic genes and toxins is necessary, as is cytotoxicity testing. 

There are 24 publicly *Achromobacter* phage genomes described in the literature (Supplemental Table S1). The first sequenced Achromophages, *Achromobacter* phage *JWAlpha* and *Achromobacter* phage *JWDelta* [13,14], are similar to N4-like phages; both are *Podoviridae*. Several more Achromophages were isolated by the same research group; however, their genomes have not been sequenced [15]. The Côte d’Ivoire Achromophage collection consists of sixteen fully characterized phages [16]. This collection was shared with our research group and used in this study. 

We have isolated and characterized thirteen lytic phages targeting *Achromobacter*, all of which are described herein. Their host range was tested in 23 *Achromobacter* clinical isolates from CF patients. Phage preparation methods based on using octanol provided high-titer phages with low endotoxin levels, appropriate for successful use in phage therapy [17]. Phage cytotoxicity was evaluated in lung epithelial cells. 

One of the bacterial isolates, *Achromobacter xylosoxidans* CF418, is a lysogen whose resident temperate phage was induced when the strain was infected with several lytic phages (described in detail below). Considerations when isolating and characterizing phages for therapeutic applications are addressed, such as the presence of prophages in host strain genomes, the presence of toxin-encoding genes in the isolated phages, and lack of functional annotation for most phage proteins. 

## 2. Results

### 2.1. Cystic Fibrosis and Achromobacter

*Achromobacter* spp. were identified as the dominant members of the microbial community in two CF fatal exacerbations. The microbial community of patient CF116′s respiratory tract was dominated by *A. ruhlandii*, which was present at a relative abundance of 98.5% based on a sputum metagenome. The clinical laboratory reported the presence of *Achromobacter* sp. and *A. xylosoxidans*. Patient CF116 was treated with the following antibiotics over a month during the acute exacerbation: ceftazidime-avibactam, doxycycline, sulfamethoxazole-trimethoprim, vancomycin, and tigecycline. A second antibiotic scheme used during the acute exacerbation included colistin, azithromycin, meropenem, imipenem-cilastatin, azithromycin, and minocycline. Patient CF116′s *Achromobacter* sp. infection was not resolved, and the patient died three months after the acute exacerbation. 

The microbial community of patient CF418′s respiratory tract was dominated by *A. xylosoxidans*, with a relative abundance of 76.7% based on a bronchioalveolar lavage metagenome obtained during an acute exacerbation. The clinical laboratory reported a rhinovirus infection one week before the acute exacerbation and chronic presence of *Achromobacter* sp. and *Pseudomonas aeruginosa*. Patient CF418 was on cardiopulmonary bypass and waiting for a lung transplant. Patient CF418 died during the acute exacerbation.

These unresolved *Achromobacter* spp. infections in two independent fatal exacerbations motivated the isolation and investigation of phages targeted against *Achromobacter* for potential future use in phage therapy. 

### 2.2. Achromobacter Clinical Isolates

*Achromobacter* spp. clinical isolates were obtained during acute exacerbations of patients CF116 and CF418. These isolates were characterized by 16S rDNA amplicon Sanger sequencing and whole genomes were obtained using Illumina and Nanopore sequencing. Henceforth, the strains are referred to as *Achromobacter ruhlandii* CF116 and *Achromobacter xylosoxidans* CF418. 

Twenty additional *Achromobacter* clinical isolates from CF patients were obtained from the UC San Diego Adult Cystic Fibrosis clinic. A reference *Achromobacter xylosoxidans* strain C54, HM-235, from a non-CF individual was obtained from the BEI collection [18]. 

### 2.3. Isolation and Characterization of Achromophages 

Fourteen Achromophages from the Côte d’Ivoire collection [16] were shared with us and used in this study. Two sequenced Achromophages were obtained from the DSMZ-German collection of microorganisms [13,19]. These are *Achromobacter* phage *JWalpha* (DSM 26830) and *Achromobacter* phage *JWDelta* (DSM 26829). Their propagation was not successful in any of the *Achromobacter* spp. strains in our collection (*n* = 23). A previous study reported the use of Achromophages in a CF patient as a phage therapy clinical intervention [11]. Access to these phages was requested but was not granted by the research group. This motivated a phage hunt strategy to construct a publicly available Achromophage library with characterized genomes. 

Thirteen lytic phages were isolated from environmental water sources, including lakes, ponds, fountains, and influents of wastewater treatment plants in San Diego, CA, USA (Table 1 and Table S2). The thirteen new *Achromobacter* phages were named using words from the Kumeyaay language spoken by native San Diegans; together, these phages are referred to as the Kumeyaay collection. 

Seven *Achromobacter* phages (*nyaak*, *kuwaak*, *ewik*, *tuull*, *maay*, *hasilly*, and *kwarr*) were isolated on *A. ruhlandii* CF116 grown in LB media supplemented with 5 mM calcium and 10 mM magnesium. It was not possible to isolate phages on lawns of *A. xylosoxidans* CF418 grown in LB media. Therefore, the subsequent isolations were carried out using both different sources of wastewater and a richer media (BHIS) to support more robust growth of the strain; using these conditions, five more Achromophages were isolated on *A. xylosoxidans* CF418 (*nyashin*, *shaaii*, *ehaak*, *emuu*, and *enyaa*). These latter five phages were able to propagate in LB media; thus, it appears that the sample source rather than the media used was the most relevant issue. 

The Kumeyaay *Achromobacter* phages genomes were sequenced (GenBank accession numbers are listed in Table 1) using the Illumina (Supplemental Table S3) and Nanopore (Supplemental Table S4) platforms. The phage genomes range in length between 33,215 bp and 50,543 bp, with GC content between 55% and 56%. Genome sizes for ten of the phages were corroborated using pulsed field gel electrophoresis (PFGE); all of the genomes were close to the 48.5 Kbp marker (Supplemental Figure S1). Phage morphology, as determined by transmission electron microscopy (TEM), showed that the phages displayed morphologies consistent with either Siphoviridae or Podoviridae (Supplemental Figure S2). 

A previously uncharacterized prophage was induced from *A. xylosoxidans* CF418 when the strain was infected with the lytic phages *nyashin*, *shaii*, *ehaak*, *emuu*, and *enyaa* (Supplemental Figures S3 and S4). 

### 2.4. Comparative Genomics of Achromobacter Phages

**Figure 1:**
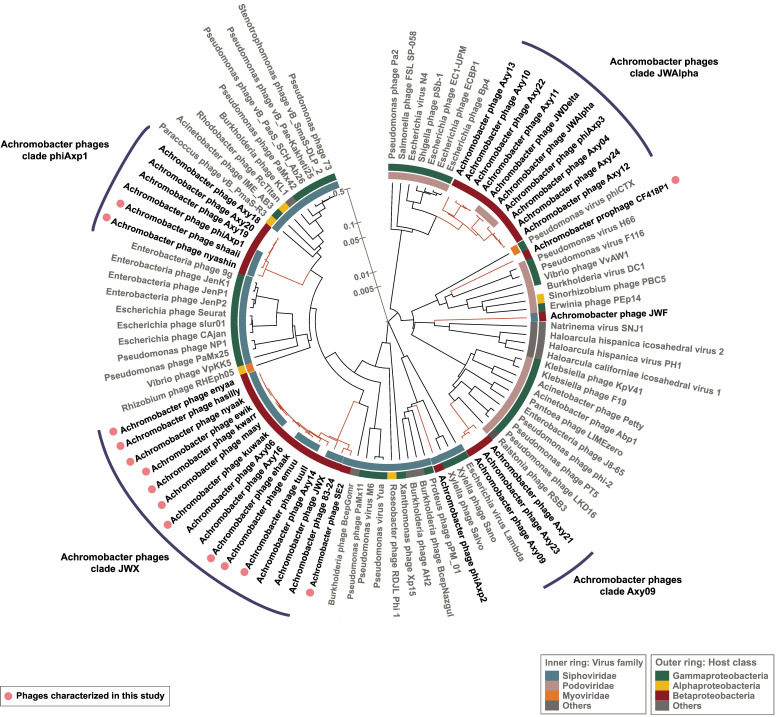
Phage proteomic tree displaying the relationships of *Achromobacter* phages. Publicly available *Achromobacter* phages, including the Côte d’Ivoire collection (*n* = 16), were compared to the Kumeyaay phages isolated in this study (*n* = 13) and the induced prophage CF418-P1. The Phage Proteomic Tree was calculated using the Viral Proteomic Tree server (https://www.genome.jp/viptree/) (accessed on March 2022). Selected phages from the ViPTree database were used to compare clades.

The ORFs encoded by each phage, numbering between 62 and 92, were predicted using Phanotate [20]. Protein-level whole genome comparisons to the Phage Proteomic Tree [21] were performed among the previously published *Achromobacter* phages (*n* = 24, Supplemental Table S1) and the Kumeyaay *Achromobacter* phages collection (*n* = 12, Table 1) using ViPTree [22] (Figure 1). The newly isolated Achromophages described herein clustered in two clades. Clade JWX (named for *Achromobacter* phage *JWX*, NC_028768.1) included phages *tuull* (aka LB2), *emuu* (aka LB7), *ehaak, kuwaak* (aka TL2), *maay* (aka LB1), *kwarr* (aka LB4), *nyaak* (aka TL1), *hasilly* (aka LB3), *wiik* (aka TL4)*,* and *ewik* (aka LB8). Clade phiAxp-1 (anchored by *A.* phage *phiAxp-1*, NC_029033.1) included Achromophages *shaaii* and *nyashin* (aka LB6). *Achromobacter* phage *83-24* (NC_028834.1) clustered in the JWX clade as well. *Achromobacter* phage *phiAxp-2* (NC_029106.1) did not cluster with the other Achromophages, clustering instead with *Burkholderia* and *Xylella* phages. A third cluster identified as JWDelta (after *Achromobacter* phage *JWDelta*, KF87094.1) was formed by Achromophages *JWAlpha*, *JWDelta*, and *phiAxp-3*. Achromophage *JWF* (NC_029075.1) was more distantly related to the rest of the Achromophages, clustering rather close to *Haloarcula* viruses (*S_G_* of 0.02), with which it shared small fragments with an identity of around 50% using tBLASTx (Supplemental Figure S5). 

**Figure 2:**
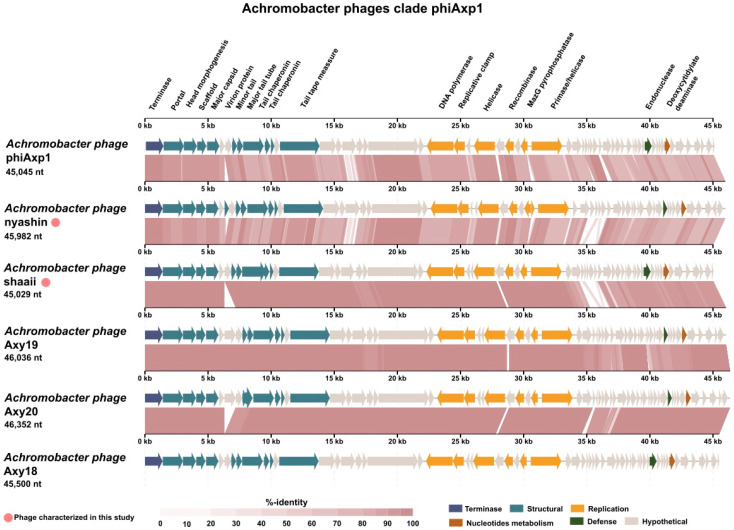
Genome comparisons of *Achromobacter* phages belonging to clade phiAxp1. All phages were aligned with the *terL* gene at the left end of the linear map. Genome maps were generated using the VIP server. Phages marked with a pink circle were isolated and described as part of our study.

In clade phiAxp1 (Figure 2), Achromophages *phiAxp-1* and *shaaii* were similar, but showed sequence variation in a 3.2 Kbp region that contained eight coding sequences. This variable region is present in the Achromophage *nyashin* as well.

**Figure 3:**
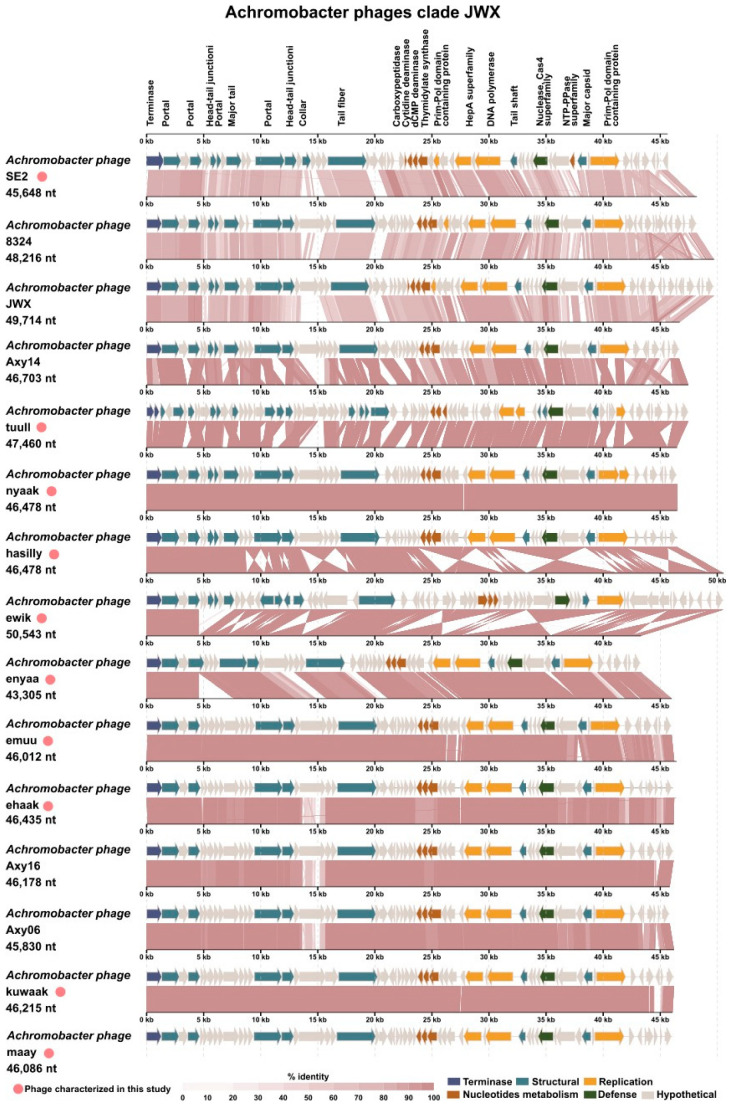
Genome comparisons of the *Achromobacter* phages belonging to clade JWX. Phages were aligned with the *terL* gene at the left end of the linear map (generated by ViPtree). Genome maps were generated using the VIP server. Phages marked with a pink circle were isolated and described as part of our study.

Small variations were observed among the phages in the clade JWX (Figure 3). Achromophages *ehaak* and *kuwaak* showed variations in a putative tail fiber protein. Achromophages *nyaak* and *hasilly* differed only by 50 nucleotides in a coding sequence that is 118 amino acids long and contains a ribbon-helix-helix domain. 

### 2.5. Achromophage Genome Annotation 

As mentioned above, Achromophages from the Kumeyaay collection encode between 62 and 92 ORFs per genome (the larger number of ORFs may be due to sequence error artifacts, due to greater number of stop codons; compare related genomes in Figure 2 and Figure 3). Genome annotations using protein comparisons based on k-mers [23] resulted in mostly hypothetical proteins, which is a common challenge in phage annotation. Further annotations using searches for conserved domains (CDD Search; [24,25,26]), hidden Markov models (HMMER Search; [27]), and artificial neural networks (PhANNs for structural proteins; [28]) followed by expert curation were used to annotate the phage genomes. Using this holistic approach, the number of hypothetical proteins was reduced. A gene encoding large terminase subunit (TerL) was identified in all these phage genomes. The small and large terminase subunits together form the packaging motor that recognizes the *pac* or *cos* site in each phage genome and packages the DNA into the phage capsids [29]. Because TerL is readily identifiable in most phages, the genome maps were oriented such that all genomes were ordered with TerL at the left edge (Figure 2 and Figure 3). A transcriptional regulator was identified in all analyzed phage genomes using the ACLAME server [30]. 

A gene for a tRNA (tRNA-Pro-TGG) was identified in nine phages, all of them members of the clade JWX. Based on sequence alignments with the *S. aureus* tRNA-Pro-TGG, the tRNA genes present in these Achromophages appear complete. The CF418-P1 prophage encodes a tRNA gene as well, in this case for tRNA-Val-AAC, which also appears complete. 

### 2.6. Annotation of Toxin-Coding and Antibiotic Resistance Coding ORFs in Achromophages

A concern in the use of phages for therapeutic application is their potential for producing toxins. Achromophages from the Kumeyaay collection have no identified toxins or virulence factors when annotated using PATRIC resources, which perform protein-level comparisons to the virulence factors database (~30,000 virulence factors reported by [31]), VICTORS (5296 virulence factors reported by [32]), and a PATRIC-curated virulence factors database (1293 virulence factors reported by [33]). A potential toxin gene was identified in the Achromophage *tuull* when the genome was annotated using the Conserved Domains Database. Further annotations using HMMER Search [27] identified an ORF of 39 amino acids as a hemolysin-type calcium binding region (HMMER identifier: A0A1Z4L446_NOSLI); 12 out of 19 amino acids were recognized as part of the toxin motif. 

No antibiotic resistance genes were identified in the phage genomes after searching using the Resistance Gene Identifier web portal (R.G.I. 6.0.2, CARD 3.2.7; most recently accessed June 2023).

### 2.7. Achromophages Lifestyle Determination 

The use of temperate phages for phage therapy is not desirable because their integration into bacterial genomes may have unexpected results, including increased virulence of the lysogen. No integrases belonging to either the tyrosine or serine families, were detected in any of the Achromophages of the Kumeyaay collection. Characterization using the PHACTS tool, which attempts to determine whether a specific phage is likely lytic or temperate [34], indicated that Achromophages *shaaii* and *nyashin* are lytic phages; the lifestyle classification for the remaining ten Kumeyaay phages was inconclusive-(Supplemental Table S5). However, experiments with the Achromophages *maay* (lysate LB1) and *Axy14*, belonging to clade JWX and with the phages *nyashin*, *Axy18*, and *Axy19* of clade phiAxp1 showed no evidence that these phages could establish lysogeny in *A. ruhlandii* CF116 and many other clinical strains.

### 2.8. Prophage CF418-P1 Induction and Characterization

**Figure 4:**
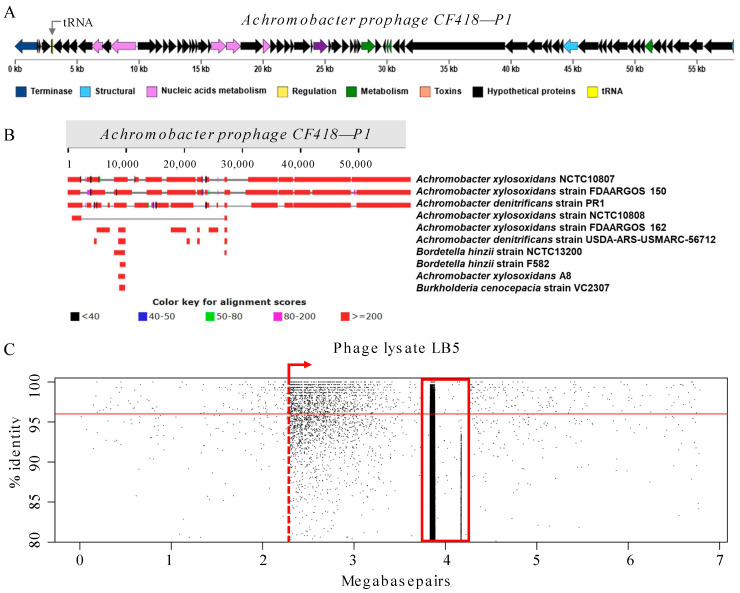
*Achromobacter* prophage was induced when the host was infected with other lytic phages. (**A**) Prophage in *A. xylosoxidans* CF418. The prophage genome was assembled from phage lysates LB5, LB7, and LB8 using SPAdes. Prophage annotation using PATRIC, CDD, HMMER, and ANNs. (**B**) Identity of *Achromobacter* phage CF418-P1 to closest bacteria genomes. (**C**) Prophage in *A. xylosoxidans* CF418. Fragment recruitment plots of phage lysate against the *Achromobacter* reference genome. The prophage region recruits reads around 3.9 Mbp (highlighted in the red vertical rectangle).

Sequencing of several of the original phage lysates (LB5, LB7, and LB8) showed that these contained multiple contigs (Supplemental Figure S3). Based on the recruitment of sequence reads from these lysates to the reference genome *Achromobacter xylosoxidans* NCTC10807 (Supplemental Figure S4), these lysates included a new prophage, which we named CF418-P1 (Figure 4A). This prophage was evidently induced to replicate when *A. xylosoxidans* CF418 was infected with the Achromophages *shaaii* and *ehaak* that were present in the LB5 lysate. The excised prophage CF418-P1 was present in lysate LB7 as well, which contained the phages *nyashin* and *emuu*; finally, the prophage was identified in lysate LB8, which also contained the Achromophages *nyashin* and *enyaa* (Supplemental Figure S3). 

Prophage CF418-P1 showed identity to *A. xylosoxidans* NCTC10807 (96.85% identity and 73% query coverage), *Achromobacter xylosoxidans* strain FDAARGOS_150 (95.70% identity and 74% query coverage), and *Achromobacter denitrificans* strain PR1 (82.77% identity and 55% query coverage) (Figure 4B). Prophage CF418-P1 is not closely related to the Achromophages from the Kumeyaay collection; it formed a separate branch in the Phage Proteomic Tree and shared six short regions with low identity (<50% using tBLASTx) to *Pseudomonas virus* H66 and *Pseudomonas virus* F116 (Supplemental Figure S6). The *A. xylosoxidans* CF418 genome was screened for prophages using PHASTER [35] (Supplemental Table S6), which predicted the one complete prophage CF418-P1 and three incomplete prophage regions.

The genome length of prophage CF418-P1 is 58,030 bp, with a GC content of 65.7%. It encodes a tyrosine family integrase and several other genes that are consistent with its temperate lifestyle (Supplemental Table S7). Nucleic acid metabolic enzymes and structural proteins were identified, as well as an endopeptidase and a peptidoglycan hydrolase. Based on the fragment recruitment plots and the gradient of the enriched bacterial sequences, prophage CF418-P1 may package segments of the host genome in its viral capsids. The fragment recruitment plots to the host genome are consistent with it having a second preferred packaging sequence (*pac* site) within the host genome where packaging initiates in a directional fashion, considering the frequency of host chromosome sequence read recruitment (Figure 4C). This suggests that this phage may be able to perform generalized transduction, albeit relatively localized to a specific region (e.g., [36]). The pattern of sequence read recruitment to the *Achromobacter* host strain genome is similar for the LB7 and LB8 lysates, but not for LB5. Based on these observations, *nyashin* may be the most likely candidate to perform generalized transduction; on the other hand, lysate LB6 contained *nyashin* and a relatively small fraction of sequences from the CF418-P1 prophage, but no other host genome sequences. The potential of LB6 to carry out generalized transduction was tested directly; see below. 

### 2.9. Host Range Determination for Achromophages 

**Figure 5:**
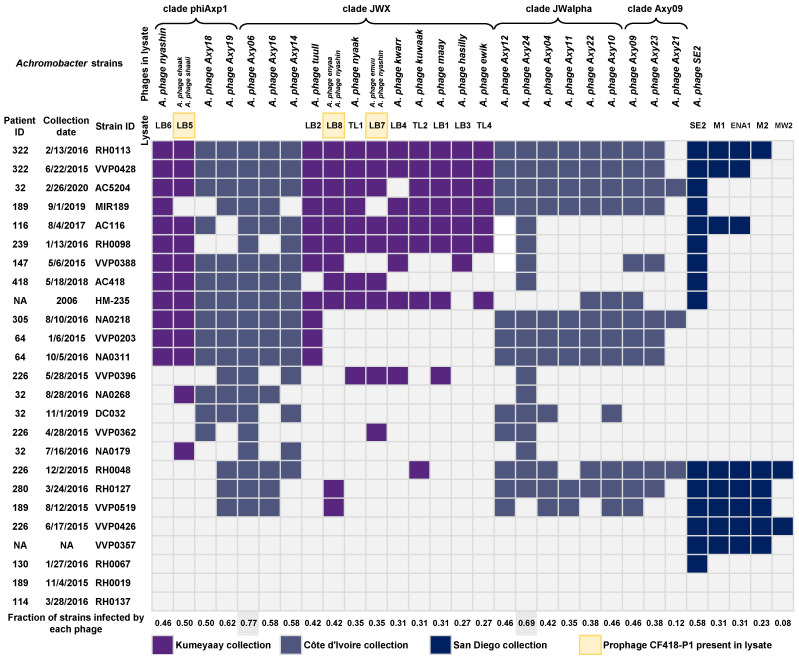
Host range of the Kumeyaay and Côte d’Ivoire Achromophages. *Achromobacter* strains isolated from the sputum of CF patients and characterized by the UCSD Medical Center clinical diagnostic laboratory were used to test host ranges. The *Achromobacter* reference strain (HM-235) was also used for testing host ranges. Strain AC116, also known as CF116, is *Achromobacter ruhlandii*. The host range was tested by performing spot tests on a lawn of the host bacteria. Lysate LB5, LB7, and LB8 contain the prophage CF418-P1 (Supplementary Figure S3). NA, not available.

The host range of Achromophages from the Kumeyaay and Côte d’Ivoire collections was tested in 24 *Achromobacter* isolates from 13 patients with CF in the San Diego clinic and the reference strain *A. xylosoxidans* HM-235 (Figure 5). Twenty out of the 25 tested *Achromobacter* isolates were lysed by at least one of the twelve phages from the Kumeyaay collection. Additional phages were isolated to target a broader set of *Achromobacter* strains; these five phages (Supplemental Table S8) are *Achromobacter* phage *SE2, Achromobacter* phage *M1, Achromobacter* phage *ENA1*, *Achromobacter* phage *M2*, and *Achromobacter* phage *MW2*. Twenty-three out of the 25 *Achromobacter* strains were infected and lysed by at least one of the isolated lytic phages. Three *Achromobacter* clinical strains were not infected by any of the isolated phages. We hypothesize that these strains carry prophages and/or phage defense systems that may prevent secondary infections by homoimmune phages or via superinfection exclusion mechanisms, thereby posing a greater challenge for the isolation of lytic phages. Achromophages *Axy06* and *Axy24*, both from the Côte d’Ivoire collection, showed the broadest host range infection at 77% and 69% of the strains tested. In contrast, Achromophage *MW2* displayed a very narrow host range, infecting only 2 out of the 25 strains tested. The *Achromobacter* phage *nyashin* was present in lysate LB6 and as one of several phages in lysates LB7 and LB8, yet these lysates differed in their host range patterns. Further study of these phage dynamics is needed in the future. 

### 2.10. Generalized Transduction Potential of the maay (LB1) and nyashin (LB6) Phages

Generalized transduction is a mechanism of horizontal gene transfer, for example of antibiotic resistance genes or toxin genes, relevant to the use of phages for therapeutic applications (e.g., [37]). As neither the bioinformatics analysis nor our observations while working with our Kumeyaay collection of lytic phages revealed any potential for establishing chromosomal integration-based lysogeny, the possibility of these phages carrying out specialized transduction of chromosomal loci was largely ruled out. However, even lytic phages such as the *E. coli* phage T4 may display a level of generalized transduction, usually less than one per million phage particles [38,39]. We tested the ability to perform generalized transduction of two phages: *maay* (LB1), representing the JWX clade, and *nyashin* (LB6), representing the phiAxp1 clade, by taking advantage of two kanamycin-resistant (Kan^R^) strains derived from our *Achromobacter ruhlandii* AC116, which serves as a host to both these phages (Figure 5). One of these putative transduction donors harbors the Kan^R^ and *gfp* genes on a broad host-range plasmid, pANT4 [40]; the other strain harbors a Kan^R^ Himar1 transposon insertion in the *fepA* gene of *A. ruhlandii* [41,42]. Briefly, the transduction assay was performed as follows: *maay* and *nyashin* lysates were grown on each of the Kan^R^ donor strains; both the *maay* and *nyashin* phages grow efficiently on AC116/pANT4 and on AC116/*fepA*::Himar1-Kan^R^ [41,42]. The donor phage lysates were mixed with each of the recipient strains at an MOI of 0.01–0.1 in liquid LB media supplemented with 10 mM magnesium sulfate and 3 mM calcium chloride (detailed in the Section 4) and incubated for 24 h. After lysis with chloroform and centrifugation to spin down cell debris, these donor lysates were mixed with the AC116 parent strain (Kan^S^) to determine the ability of the phages to perform transduction. A donor lysate grown on AC116 served as the negative control. The transduction was performed using a protocol similar to that used for *E. coli* phage P1 transduction [43], as described in Section 4. Phages were allowed to absorb to recipient cells for 30 min at 30 °C. The mixture of donor phage lysate and recipient cells was then concentrated, and the pellet was resuspended in 0.3 mL of LB with 0.1 M Na-citrate to prevent further phage infection. These mixtures were further incubated for 60 min to allow expression of the Kan^R^ gene, then concentrated and spread onto LB plates containing 60 μg/mL kanamycin. The plates were incubated for 48 h at 37 °C. No Kan^R^ transductants were observed after 48 h, for a calculated frequency of transduction of less than one transductant per 8 × 10^7^ cells.

### 2.11. Preparation of Achromobacter Phages Lysates for Therapeutic Applications

**Figure 6:**
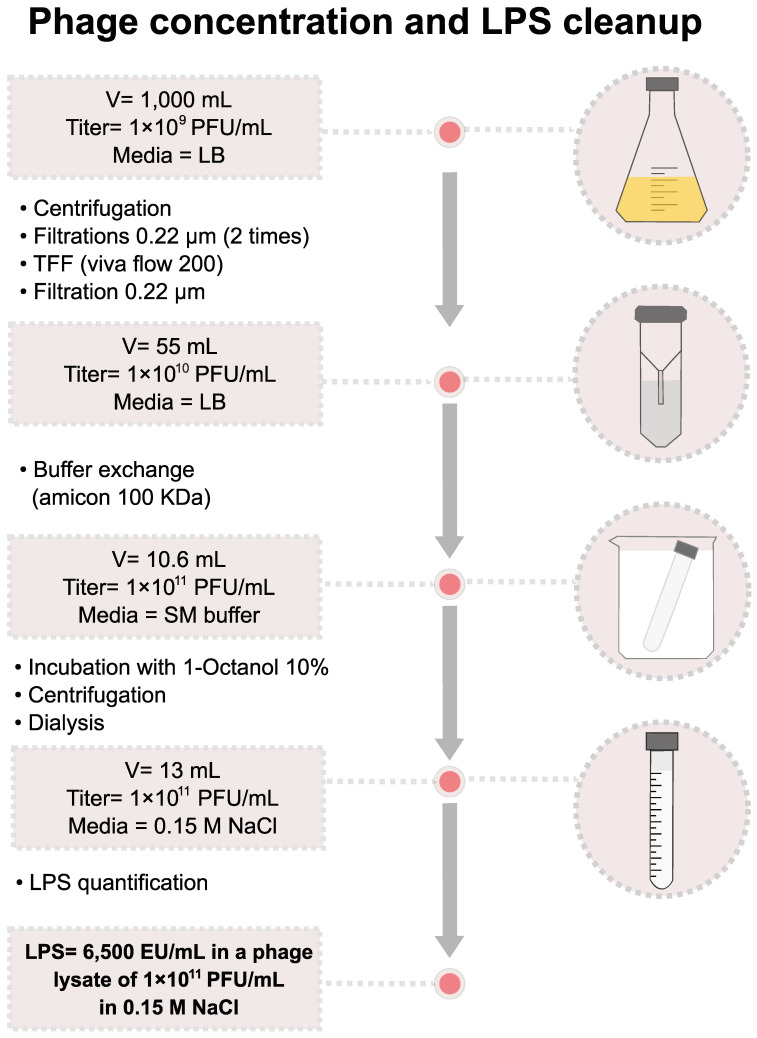
Protocol for *Achromobacter* phage *nyaak* high-titer lysate production and endotoxin removal. The *Achromobacter* phage *nyaak* was produced in *Achromobacter ruhlandii* CF116. Final endotoxin units were measured with the Endozyme II kit from Biomerieux.

Phage production for therapeutic application requires a high titer phage lysate with a low concentration of lipopolysaccharides (also known as LPS, lipoglycans, or endotoxins). Thus, the ideal production method should preserve and preferably concentrate the phage titer, should be fast and relatively inexpensive, and should avoid the use of toxic compounds. Our method, illustrated using the Achromophage *nyaak*, is shown in Figure 6. The final preparation has a titer of 1 × 10^11^ PFU mL^−1^ in a 0.15 M NaCl solution, with the concentration of lipopolysaccharides being 6500 EU mL^−1^. 

### 2.12. Evaluation of Phages Cytotoxicity in Lung Epithelial Cells

**Figure 7:**
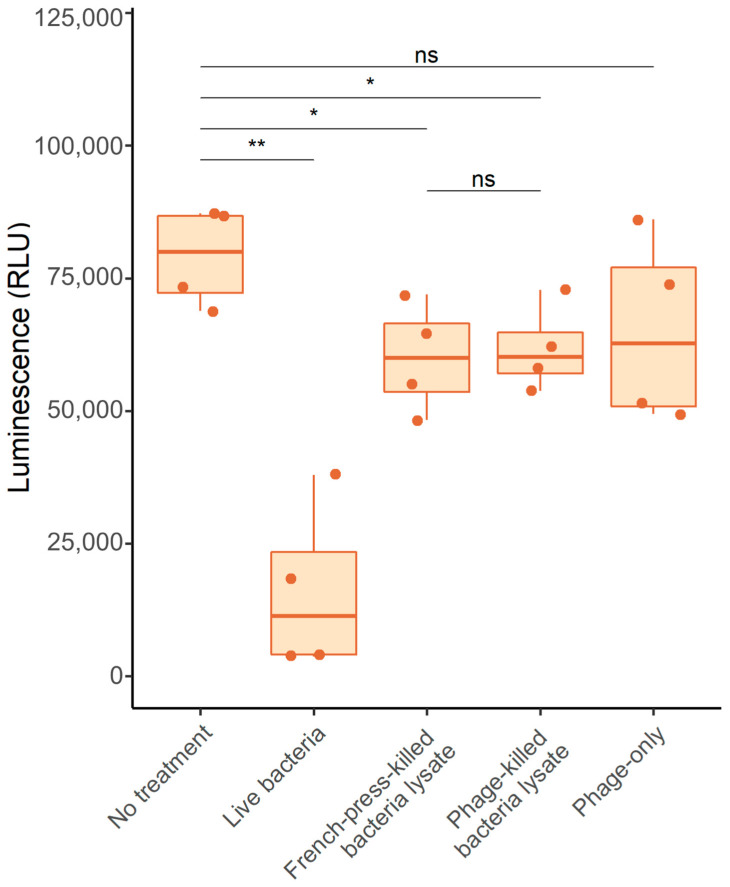
Cytotoxic effects of bacteria and/or phages on A549 cells. The *Achromobacter* phage *nyaak* and *Achromobacter ruhlandii* CF116 were used in the experiments. Ns = not significant, * *p*-value < 0.01, ** *p*-values < 0.001.

Two consequences of lytic infection may result in toxicity to eukaryotic cells; one is potential toxicity of the phage lysate itself, possibly due to insufficient removal of endotoxin, while a second is that lytic infection may induce gene expression and replication of a prophage within the strain targeted by the phage, which may result in production of an exotoxin such as STX, as seen in the case of the Shiga toxin-producing *E. coli* [44]. Therefore, we evaluated the cytotoxicity to A549 lung epithelial cells of a phage lysate or of lysed *Achromobacter*, either by phage infection or, as a control, by a cell lysate made by disrupting cells using a French press (Figure 7). The viability of A549 cells was assessed by measuring ATP production using a luminescence assay (see Section 4). *Achromobacter* bacteria added to A549 cells in culture severely reduced viability compared to untreated control cells. In contrast, neither phage-killed bacterial lysates nor French press-killed bacterial extracts caused appreciable cytotoxicity (reduction in ATP levels) to A549 cells, and we found no significant difference between the bacterial preparations lysed using the two different methods. This indicated that the tested phage did not induce the production of toxic factors while killing the bacterial cells. Phage lysates from which endotoxin was removed were applied, and again no cytotoxicity was observed; in fact, there was no significant difference between phage-lysed cells and French press-lysed cells (Figure 7).

## 3. Discussion

### 3.1. Hunting for Achromophages 

The availability of sequenced *Achromobacter* phages was limited at the start of our study, with twenty-four *Achromobacter* phage genomes [14,15,16,45,46,47] available in public databases. 

Phages capable of killing *Achromobacter* spp. were isolated from water samples collected around San Diego, CA, USA. Phages that infected and replicated in *A. ruhlandii* CF116 were readily isolated within the first two weeks of the search; in contrast, it took six months to isolate phages that infected *A. xylosoxidans* CF418. The most likely reason for this is the presence of prophages in *A. xylosoxidans* CF418, which may have provided immunity or protection by restriction, superinfection exclusion, or other mechanisms against invading phages. The host strains used in phage hunts fundamentally affect whether such phage screens are relatively easy or more challenging. The prophage CF418-P1, described below, was induced to lytic growth by co-infection with several lytic phages. Three other prophages identified in the CF418 strain’s genome were not induced to lytic growth, yet may inhibit infection or replication of the host via DNA modification or superinfection exclusion, via immunity and/or other defense mechanisms of prophages against incoming lytic phages [48]. Three of the 23 *Achromobacter* spp. strains in our collection in this study were not lysed by any of the thirteen Kumeyaay lytic phages described herein nor any of the sixteen Côte d’Ivoire phages isolated by Essoh and colleagues [16]. Because of these limitations on phage isolation, access to multiple publicly available phage collections and cooperation among research groups are critical for the advancement of the phage therapy field specifically and phage biology in general. 

### 3.2. Induction of Cryptic Prophages by Lytic Phage Replication 

During the isolation of lytic phages the CF418-P1 prophage was induced from *A. xylosoxidans* CF418. Whether the induction of this prophage promotes expression of any toxins was not determined. The genome of the induced prophage was detected in the lysates of the infecting phages and present as a complete genome that could be circularize. In addition, evidence for apparent generalized transduction was observed in the case of prophage CF418-P1: sequence reads showed the packaging of segments of the host genome in its viral capsids, including evidence for a *pac*-like site in the chromosome where unidirectional DNA packaging was initiated (Supplemental Figure S4). This phenomenon has been observed in other Achromophages, such as phage α [49]. Cryptic *Achromobacter* prophage induction mediated by superinfection with a related phage has been reported before [50]. This phenomenon may be related to phage induction of chromosomal islands [51,52] or due to SOS induction by some phage replication intermediates. Instances of specialized transduction have been observed by a *Pseudomonas* prophage that is somewhat related to *Achromobacter* prophage CF418-P1 [53]. We tested the generalized transduction potential of phages *maay* and *nyashin*, representative of the JWX and Axp1 *Achromobacter* phage clades, respectively, and found fewer than one transductant per 8 × 10^7^ cells, the limit of detection of the assay. 

### 3.3. Endotoxin Removal from Phage Lysates for Therapeutic Applications

The proposed method for endotoxin removal from phage lysates was successful both with respect to the phage titer recovered and the low amount of endotoxin left in the preparation. Phages for therapeutic applications are often delivered or applied as a 1 × 10^9^ PFU mL^−1^ solution. The phage stock presented in this work had a final concentration of 65 EU mL^−1^ when diluted to a concentration of 1 × 10^9^ PFU mL^−1^. The FDA-recommended maximum dose of endotoxin units (EU) in a solution administered intravenously is 5 EU per kilogram of body weight per hour. Up to 6 mL of the phage stock of 1 × 10^9^ PFU mL^−1^ that contains 65 EU mL^−1^ can be applied to a patient (average weight of 80 kg) every hour. The dosing of phage preparations in patients is empirical, and more efforts towards understanding phage pharmacokinetics are needed [54]. While the residual amount of 1-octanol may be concerning, it is noteworthy that 1-octanol is metabolized in the human body by class IV alcohol dehydrogenases [55,56,57]. This is an advantage of using 1-octanol for LPS removal rather than phenol extraction, although other methods such as hexafluoroisopropanol are available [58].

### 3.4. Identification of Toxin and Antibiotic Resistance Genes in Phage Genomes

Identification of phage toxins based on genome sequencing is challenging [59]. A toxin motif was identified in the Achromophage *tuull*, making this phage unsuitable for therapeutic use. No known toxins were identified in the other Kumeyaay phage collection. Because most phage ORFs were annotated as hypotheticals, it is formally possible that uncharacterized toxins may be present in these and other phages intended for therapeutic use. A more sensitive method to indicate the safety of phages for potential therapeutic use is to test the cytotoxicity of the lysates in eukaryotic cell lines [60,61], as we have described for one of our phages, and/or whether they are well-tolerated in animal models. 

In addition, we screened against CARD for the presence of antibiotic resistance genes in the Kumeyaay phage genomes and found none.

### 3.5. Evaluation of Phage Cytotoxicity in Lung Epithelial Cells

While phage particles seem not to be toxic to human cells, this needs to be tested in the context of the therapeutic lytic phage and the target bacteria, as there are several unknown factors in the lytic killing of bacteria that can potentially affect human cells. Lytic infection with a therapeutic phage may induce other phages in the target cell and cause damage to eukaryotic cells. To evaluate this, we developed an in vitro assay to test the cytotoxicity of phage-killed bacterial cells. We did not observe any decrease in A549 viability in the presence of phage-killed bacteria compared to mechanically-lysed bacteria, which suggests that this phage is not toxic. 

### 3.6. Beyond Phage Hunting

Alternative solutions to phage isolation are the engineering of phages to expand their host range [62], the use of phage-derived tails (aka tailocins) for therapeutic applications [63], and the use of phage-derived endolysins [64]. Each of these methods has advantages and disadvantages. Clearly, the therapeutic use of native phages will continue long into the future, either alone and/or in combination with antibiotics [8,65,66]. 

## 4. Materials and Methods

### 4.1. Cystic Fibrosis Metagenomes

Informed consent was obtained from patients CF116 and CF418. This study was approved by UCSD (HRPP 081510) and San Diego State University (IRB#1711018R). For patient CF116, a sputum sample was collected in a sterile cup after sputum induction. For patient CF418, a bronchioalveolar lavage sample (BAL) was collected. From the BAL or sputum samples, 500 µL were transferred to a cryovial. To isolate total community DNA, the protocol started with cell lysis by submerging the tube in a dry ice and ethanol bath for 5 min then transferring to a water bath at 100 °C for 5 min; this process was repeated three times. The sample was transferred to the bead-containing tube included in the Qiagen power soil DNA extraction kit (catalog number 12888-100) and homogenized by shaking for 45 min. The rest of the Qiagen power soil DNA extraction kit protocol was followed. Ten nanograms of DNA were used for Nextera library preparation. Libraries were sequenced on the Illumina MiSeq platform. 

### 4.2. Achromobacter Strains 

*Achromobacter* clinical isolates from patients CF116 and CF418 were phenotypically characterized by the UCSD clinical laboratory and grown in Remel blood agar. Both clinical isolates can grow in tryptone yeast extract glucose medium (TYG), in lysogeny broth (LB), and in supplemented brain heart infusion broth (BHIS). The BHIS media was supplemented with the following per liter: 5 mg hemin, 1 mg menadione, 5 g yeast extract, 50 mg L-cysteine HCl, 120 mg MgSO_4_, and 50 mg CaCl_2_. Capsule formation was observed in the liquid culture, which is characteristic of many pathogenic *Achromobacter* strains. Each clinical isolate was cultured in 3 mL of liquid LB media (tryptone 10 g/L, yeast extract 5 g/L, sodium chloride 10 g/L) at 37 °C for 16 h, and cells were pelleted by centrifugation and resuspended in molecular grade water. Total DNA was extracted using a Qiagen (Hilden, Germany) blood and tissue kit (Cat. No. 69504). PCR to amplify 16S rDNA was performed on each isolate using 27F and 1492R primers, and the ~1500 bp amplicons were sequenced by Sanger. The resulting sequences were compared to the NCBI nr database using online megablast. The closest hit was to *A. xylosoxidans* for both isolates. A total of 400 nanograms of DNA were used for whole-genome sequencing on the Nanopore platform, and genomes were sequenced in the Illumina platform as well. Whole-genome analysis of both isolates determined the strains as *Achromobacter xylosoxidans* CF418 and *Achromobacter ruhlandii* CF116. 

### 4.3. Phage Hunting 

Aqueous samples (lake water, pond water, and fountain water, locations provided in Supplemental Table S2) were collected and filtered through a 0.22 µm filter (made of polyvinylidene fluoride, Fisher Scientific (Hampton, NH, USA) Cat. No. SLGVR33RS) and stored at 4 °C. Samples of influent from wastewater treatment plants were stored at 4 °C, then a 50 mL aliquot was centrifuged at 4000 RPM for 10 min to pellet debris and the supernatant was filtered with a 0.45 µM filter (made of polyvinylidene fluoride, Fisher Scientific Cat. No. SLHVR33RS). Chloroform was added to 5% *v*/*v* for long term storage at 4 °C. 

Phage isolation for *A. ruhlandii* CF116 was performed based on PhagesDB [67] protocols with the addition of 10 mM MgSO_4_ and 5 mM CaCl_2_ to bacterial cultures and top agar. Plates were incubated at 37 °C overnight and examined for phage plaques. Individual plaques were streaked onto a top agar plate for purification of each phage; plaque purification was repeated sequentially a total of three times. After plaque purification, 3.5 mL of each phage lysate were prepared by transferring a purified plaque into a logarithmically-growing bacterial sub-culture and incubated overnight at 37 °C. This small lysate was then used to prepare a larger stock of phage lysate (50 mL) (Supplemental Table S2).

Phage isolation in *A. xylosoxidans* CF418 was performed as follows. Frozen glycerol (12.5% final concentration) stock was streaked onto BHIS plates and incubated at 37 °C for 24 h in an anaerobic chamber. Individual colonies were then cultured at 37 °C for 24 h in 3–5 mL BHIS broth. Four mL of BHIS top agar, 200 µL host overnight culture, and processed influent (0.1–1 mL) were combined and poured as top agar overlays on BHIS plates. Individual plaques were then selected by streak-isolation with a toothpick to a new top agar plate. The phages were passaged at least three times until only one plaque phenotype was visible. The phage lysates were stored at 4 °C and in 25% glycerol stocks. 

### 4.4. Transmission Electron Microscopy (TEM) of Phages

Phage preparations were stained with uranyl acetate for transmission electron microscopy as follows [68]. Glow-discharged 300 mesh copper grids coated with carbon and formvar were overlaid with drops (30 µL) of purified phage samples for 3 min. Salts were removed from the buffer by rinsing the grids three times with drops of water (20 µL). Next, the grids were negatively stained with uranyl acetate (0.5 %) for 15 s, dried, and examined using a FEI Tecnai T12 TEM (FEI, Hillsboro, OR, USA) operating at 120 kV at the SDSU Electron Microscopy Facility. Micrographs were taken with an AMT HX41 side-mounted digital camera (Advanced Microscopy Technique, Woburn, MA, USA) (Supplemental Figure S2). A lysate titer of 1 × 10^10^ PFU/mL is ideal.

### 4.5. Determination of Phage Genome Size by Pulsed Field Gel Electrophoresis (PFGE) 

Two hundred and fifty µL of pure phage resuspended in SM buffer was added to an equal volume of 1.6% low-melting (LM) agarose prepared in molecular grade 0.02 µm filtered water. The phage concentrations in the starting suspensions are shown in Supplemental Figure S1C. Before mixing, the LM agarose was placed in a 50 °C water bath for 20 min to avoid heat damage to the phage particles. The mix was immediately distributed in individual 75 µL wells of plug molds and allowed to solidify for 20 min at 4 °C. A small suction bulb was used to suck the plugs out of the molds and place them in TE (10 mM Tris-HCl, 0.1 mM EDTA, pH 7.5) (2 mL of solution/3 plugs), using flat-bottomed tubes to avoid breaking the plugs during the procedure. Contamination with free DNA (released from cells upon lysis) was minimized by incubating the plugs in a solution containing 1 µg mL^−1^ of DNase and 1X DNase buffer in TE, at 37 °C for 1 h. The liquid was then removed and the plugs were transferred to a new tube containing ESP (0.5 M EDTA, pH 9, 1% N-laurylsarcosine, 1 mg mL^−1^ proteinase K) then incubated at 50 °C overnight (ON). In order to inactivate the proteinase K, the plugs were transferred to a new tube containing PMSF solution (1 mM PMSF, 20 mM Tris-HCl, pH 8, 50 mM EDTA) and incubated for 1 h at room temperature (RT) on a tube rocker under gentle agitation. The plugs were washed six times with TE; for the first wash, the plugs were transferred to a new tube and left ON at RT under gentle agitation. The subsequent five washes were performed for 30 min each without exchanging tubes. Using low EDTA TE (10 mM Tris and 0.1 mM EDTA), six extra washes of 30 min each were performed in the same conditions as the previous ones. After this step, the plugs were maintained in low EDTA-TE at 4 °C until the agarose gel was prepared. The 0.22 µm filtered 0.5X TBE was kept in the PFGE machine until the temperature reached 14 °C. After this step, the plugs were cut in half and placed in the wells of the 1% PFGE Agarose (BioRad) in the same filtered TBE. The wells were closed with the melted agarose used to make the gel, which was kept in a 50 °C water bath. The gel was left at room temperature for ~5 min until the agarose polymerized, then loaded in the PFGE apparatus. The electrophoresis conditions were automatically set by the instrument using the option “auto algorithm” and adding the range of the standard markers (in this case, from 15 Kb to 300 Kb). The gradient selected was 6 V/cm, the time was 23:52 h, the included angle was 120°, the initial switch time was 1.19 s, and the final switch times were 26.29 s. The MidRange PFG marker (New England Biolabs, Ipswich, MA, USA) and T4 phages were used as size standards (Supplemental Figure S1B). 

### 4.6. Determination of Phage Host Range

The host range of the isolated phages was tested against a collection of 24 *Achromobacter* strains isolated from sputum of cystic fibrosis patients at the UCSD CF clinic and the reference strain *Achromobacter xylosoxidans* HM-235. Host range was tested by spotting 10 µL of phage lysate in top agar of a lawn of the bacteria. Lysis was evaluated after 16 h of incubation at 37 °C. 

### 4.7. Isolation of Phage DNA for Sequencing

Fifty milliliters of each phage lysate were saved prior to chloroform treatment to minimize the amount of free bacterial DNA. The phage DNA isolation protocol [69] (Supplemental Figure S7) consisted of phage lysate filtration through a 0.22 μm filter followed by a first DNase and RNase treatment, PEG precipitation, a second DNase and RNase treatment, proteinase K treatment, and the “popping” of viral particles by passing through the resin from the Promega Wizard DNA clean-up system (Cat. No. A7280). The resulting DNA pellet was resuspended in molecular grade water.

### 4.8. Illumina Sequencing of Phages

Ten nanograms of phage DNA were used for library preparation using the Swift ACCEL-NGS 1S PLUS (Cat. No. 10024) with sixteen cycles of PCR amplification and an additional bead cleanup step (AMPure XP, Beckman-Coulter (Indianapolis, IN, USA), Cat. No. A63881) with a proportion of 0.85X AMPure beads at the end of the library prep to remove sequencing adapters. Libraries were pooled and sequenced in the Illumina platform MiSeq as paired ends with 300 cycles. The number of reads obtained per phage was between 400 reads and 5 million (Supplemental Table S3). In the case of phage *SE2*, isolated phage DNA was sent to the MiGS Center, Pittsburgh, PA, USA, for library preparation and sequencing.

### 4.9. Nanopore Sequencing of Phages

Phage DNA (3 ng to 400 ng) was sequenced using the Nanopore minIon in a single flow cell R9 with barcoding; between 200 and 5000 reads were obtained per phage (Supplemental Table S4). 

### 4.10. Assembly of Phage Genomes

Paired end reads were filtered for quality using PRINSEQ++ [70] (--entropy = 0.5 --trim_qual_right = 15 --trim_qual_left = 15 --trim_qual_type mean --trim_qual_rule lt --trim_qual_window 2 --min_len 30 --min_qual_mean 20). A subsample with a replacement of 50,000 or 100,000 reads per phage was used for de novo assembly with SPAdes using the parameter --only-assembler [71]. Attempts to assemble using all reads resulted in fragmented phage genomes. Assembly graphs (.fastg files) were inspected using BANDAGE [72]; while in most cases a complete genome was obtained in a single contig graph, for certain phages, e.g., phage *tuull*, several contigs were concatenated to obtain the complete genome, as several graph paths were obtained from the assembler. Phage lysates propagated in *Achromobacter* CF418 had more than one complete phage genome in the assembled contigs; specifically, a second phage genome was identified in lysates LB5, LB7, and LB8 (Supplemental Figure S3). The same “common” contig was identified as the temperate prophage in *Achromobacter* CF418, CF418-P1. This indicated that infection with several distinct lytic phages, namely, *ehaak* and *shaii* in lysate LB5, *nyashin* and *emuu* in lysate LB7, and *nyashin* and *enyaa* in lysate LB8, resulted in the induction of the resident prophage CF418-P1. In order to more easily compare phage genomes, the genome maps were sorted by placing the TerL terminase gene at the left end of the linear maps after using the program circulaline [73]. 

### 4.11. Annotation of Phage Genomes 

Phage genomes were annotated in PATRIC [33] using the PHANOTATE algorithm, which is optimized for phage gene calling [20]. Using this approach, most of the protein annotations were hypothetical. To improve annotations, the phage protein sequences were further analyzed using the conserved domains database (CDD) [24,25,26], HMMER search [27,74], and the machine learning-based ensemble of artificial neural networks PhANNs, which is trained to predict phage structural proteins [28]. Expert manual curation of each phage annotation was performed. Genome maps were obtained using Easyfig [75]. Phage genomes were screened for antibiotic resistance genes using the Resistance Gene Identifier web portal (RGI 6.0.2, CARD 3.2.7) [76].

### 4.12. Comparative Genomics of Achromophages

Available *Achromobacter* phage genomes (*n* = 24) and phage genomes isolated in this study (*n* = 13) were compared using the Phage Proteomic Tree approach [52] using ViPTree [22], as shown in Figure 1. The linear genome alignments shown in Figure 2 and Figure 3 were generated using ViPTree genome alignment view, which uses tBLASTx normalized scores (S_G_) with a cutoff of E-value < 0.01. 

### 4.13. Testing Potential for Generalized Transduction of Phages Maay and Nyashin

We tested the ability to perform generalized transduction of two phages: *maay* (LB1), representing the JWX clade, and *nyashin* (LB6), representing the phiAxp1 clade, by taking advantage of two Kan-resistant strains derived from our *Achromobacter ruhlandii* AC116 strain, which serves as a host to both these phages (Figure 5). One of these potential transduction donor strains harbors the Kan^R^ and *gfp* genes on a broad host-range plasmid, pANT4 [40], while the other strain harbors a Himar1-Kan^R^ insertion in the *fepA* gene of *A. ruhlandii* [41,42,77,78]. Briefly, the transduction assay was performed as follows: *maay* and *nyashin* lysates were grown on each of the donor strains (AC116/pANT4 and AC116/*fepA*::Kan^R^) [41,42]. *Maay* or *nyashin* phage lysates were mixed with each of the donor strains at an MOI of 0.01–0.1 in liquid LB media supplemented with 10 mM Mg_2_SO_4_, 3 mM CaCl_2_, 5 mM Na_2_HPO_4_-7H_2_O, 2.26 mM KH_2_PO_4_, 0.9 mM NaCl, 1.9 mM NH_4_Cl_2_, and 0.01% glucose, then incubated with shaking for 24 h at 37 °C. Chloroform was added to these phage lysates to release all phages that may not have lysed cells on their own, and were then centrifuged for 15 min at 3500 rpm to spin down the cell debris. For transduction, the donor phage lysates were mixed with the AC116 parent strain (Kan^S^) to determine the ability of the phages to perform transduction. A donor lysate grown on AC116 served as the negative control. The transduction was performed using a protocol similar to that used for *E. coli* phage P1 transduction as described in [43]. Briefly, potential donor phage lysates (10 mL) were mixed with recipient cells (8 × 10^7^ cells in 0.3 mL) and allowed to absorb to recipient cells for 30 min at 30 °C. The mixture of donor phage lysate and recipient cells was concentrated and the pellet was resuspended in 0.3 mL of LB with 0.1 M Na-citrate, to prevent (or reduce) further phage infection. These mixtures were further incubated for 60 min at 37 °C to allow expression of the Kan^R^ gene, then concentrated and spread onto LB plates containing 60 μg/mL kanamycin sulfate and incubated for up to 48 h. None of the combinations of donor phages and recipient strains yielded any transductants; hence, the calculated transducing frequency was less than one in 8 × 10^7^ cells, the limit of detection.

### 4.14. High-Titer Phage Production and Endotoxin Removal and Quantification

Lysate from the *Achromobacter* phage *nyaak* (1 L at 1 × 10^9^ PFU mL^−1^) was produced in LB media supplemented with 10 mM MgSO_4_ and 5 mM CaCl_2_. The clean-up procedure is illustrated in Figure 6. The lysate was centrifuged to remove bacterial debris, then filtered twice through a 0.22 µm filtering cup to remove remaining bacteria cells. The remaining bacterial cells were not disrupted by chloroform treatment to avoid an increase in LPS in the solution. Concentration was performed in a tangential flow filtering apparatus (Viva Flow 200, Sartorius, with a molecular weight cut-off of 100 kDa) to 55 mL, followed by filtration through a 0.22 µm filtering cup. The phage titer after this step was 1 × 10^10^ PFU mL^−1^. Phages are more stable in SM buffer than in LB; thus, buffer exchange and further concentration were performed via ultrafiltration using a 100 kDa cellulose membrane (Amicon Ultra-15, Millipore (Burlington, MA, USA)) After exchanging the media from LB to SM buffer, the concentrated volume was 10.6 mL with a phage titer of 1 × 10^11^ PFU mL^−1^. Endotoxin (lipopolysaccharides) was removed through incubation with 10% 1-octanol as described previously [79,80]. The residual 1-octanol was reduced using dialysis. The concentrated phage lysate was placed in dialysis tubing with a 100 Da molecular weight cut-off, and four exchanges were performed against 0.15 M NaCl. The lysate volume after dialysis was 13 mL, with a titer of 1 × 10^10^ PFU mL^−1^ in 0.15 M NaCl. Residual lipopolysaccharides were quantified as endotoxin units (EU) using recombinant factor C-based fluorescence detection (EndoZyme II, Biomérieux (Marcy-l’Étoile, France)), which determined that 6500 EU mL^−1^ were present in the last concentrated and octanol-cleaned lysate dialyzed into 0.15 M NaCl. The phage stock was stored at 4 °C in the dark. This procedure was adapted from previous reports [81] and is a CsCl-free alternative method for phage therapy applications.

### 4.15. Evaluation of Phage Cytotoxicity in Lung Epithelial Cells

Confluent monolayers of A549 cells were grown in a 96-well plate. Treatments were applied in volumes of 50 µL. For treatment with live bacteria, an overnight culture of *A. ruhlandii* CF116 was used containing 5 × 10^9^ CFU mL^−1^. For the French-press killed treatment, 5 mL of *A. ruhlandii* CF116 overnight culture (5 × 10^9^ CFU mL^−1^) were passed through a French press twice and 50 µL of the resulting extract were used for treatment. A subculture from an overnight culture of *A. ruhlandii* CF116 was infected with the *Achromobacter* phage *nyaak* and incubated until lysis was observed (~8 h); 50 µL of this lysate were used for treatment (see Figure 7). A lysate from the *Achromobacter* phage *nyaak* from which endotoxin had been removed was used for the phage-only treatment. All treatments were added to A549 cell monolayers of confluent cells. The viability of the A549 cells was measured using a luminescent cell viability assay 24 h post-treatment (CellTiter-Glo, PROMEGA (Madison, WI, USA)) according to the manufacturer’s protocol. Cell viability was expressed as relative luminescence units. Four biological replicate experiments were performed for each treatment. 

## Figures and Tables

**Table 1:** *Achromobacter* phages isolated in this study. Etymologies are from the Kumeyaay language. All isolation sources are from San Diego, CA, USA. Coding sequences, tRNAs and repeat regions were obtained from PATRIC. GenBank accession numbers are listed.

Phage Name (GenBank Accession Number)	Genome Length (bp)	GC Content	CDS	tRNA	Repeat Regions	Clade	Host	Phage Name Etymology
*nyashin* (OQ817840)	45,982	56.31	68	0	2	phiAxp1	*A.ruhlandii* CF418	*nyashin–*today
*shaaii* (OQ817841)	45,029	56.11	63	0	0	phiAxp1	*A.ruhlandii* CF418	*shaaii*–buzzard
*nyaak* ( OQ817839)	46,478	55.77	66	tRNA-Pro-TGG	2	JWX	*A. ruhlandii* CF116	*nyaak*–east
*kuwaak* (OQ817836)	46,215	56.19	66	tRNA-Pro-TGG	0	JWX	*A. ruhlandii* CF116	*kuwaak*–south
*ewik* (OQ817842)	50,543	55.75	83	tRNA-Pro-TGG	37	JWX	*A. ruhlandii* CF116	*ewik*–west
*tuull* (OR396896)	47,460	55.79	92	tRNA-Pro-TGG	15	JWX	*A. ruhlandii* CF116	*tuull*–north
*maay* (OQ817838)	46,086	56.31	62	tRNA-Pro-TGG	2	JWX	*A. ruhlandii* CF116	*maay*–sky
*hasilly* (OQ817843)	46,478	55.77	65	tRNA-Pro-TGG	2	JWX	*A. ruhlandii* CF116	*hasilly*–sea
*ehaak* (OQ817833)	46,435	56.17	64	tRNA-Pro-TGG	0	JWX	*A.ruhlandii* CF418	*ehaak*–raven
*emuu* (OQ817834)	46,012	55.86	62	tRNA-Pro-TGG	2	JWX	*A.ruhlandii* CF418	*emuu*–mountain sheep
*enyaa* (OQ817835)	43,305	55.51	64	tRNA-Pro-TGG	0	JWX	*A.ruhlandii* CF418	*enyaa*–sun
*kwarr* (OQ817837)	33,215	55.59	73	0	6	JWX	*A. ruhlandii* CF116	*kwarr*–red earth to paint the body
*SE2* (OQ817844)	45,648	55.88	55	tRNA-Pro-TGG	0	JWX	*Achromobacter* sp. VPP0426	
*prophage* CF418-P1 (OQ817832)	58,030	65.63	73	tRNA-Val-AAC	2		*A.ruhlandii* CF418	

## Data Availability

Bacterial strains and bacteriophages are available upon request from Anca Segall. Sequence data for the bacteriophages is deposited at GenBank (NCBI).

## Supplementary Materials

The following supporting information can be downloaded at: https://www.mdpi.com/article/10.3390/v15081665/s1, Figure S1: Determination of phage genome sizes by PFGE; Figure S2: *Achromobacter* phages transmission electron microscopy; Figure S3: Prophage induced in *Achromobacter* CF418 when infected with additional lytic phages; Figure S4: Prophage induced in *Achromobacter* CF418 when infected with lytic phages; Figure S5: Phage Proteomic Tree alignment visualization of *Achromobacter* phage JWF and its closest relatives; Figure S6: Genome comparison of Achromobacter prophage CF418-P1 and its closest phage relatives; Figure S7: Protocol for phage propagation and DNA extraction for genome sequencing; Table S1: *Achromobacter* lytic bacteriophage genomes in the literature (n = 24); Table S2: Phage lysates and phage genomes names; Table S3: Illumina sequencing information for Achromophages; Table S4: Achromophages Nanopore sequencing information; Table S5: Lifestyle prediction for Achromobacter phages; Table S6: Predicted prophages in *Achromobacter xylosoxidans* CF418; Table S7: Prophage CF418-P1 genome annotation; Table S8: Isolated Achromophages for broader host range.
