# Supplementary material for: Compounding Achromobacter Phages for Therapeutic Applications
Source: Viruses. 2023 Jul 30;15(8):1665. doi: 10.3390/v15081665 (PMC10457797; doi:10.3390/v15081665)
Supplement: Supplementary file 1 [file viruses-15-01665-s001.zip › viruses-2470797-supplementary.pdf]

Supplemental Figures and Tables

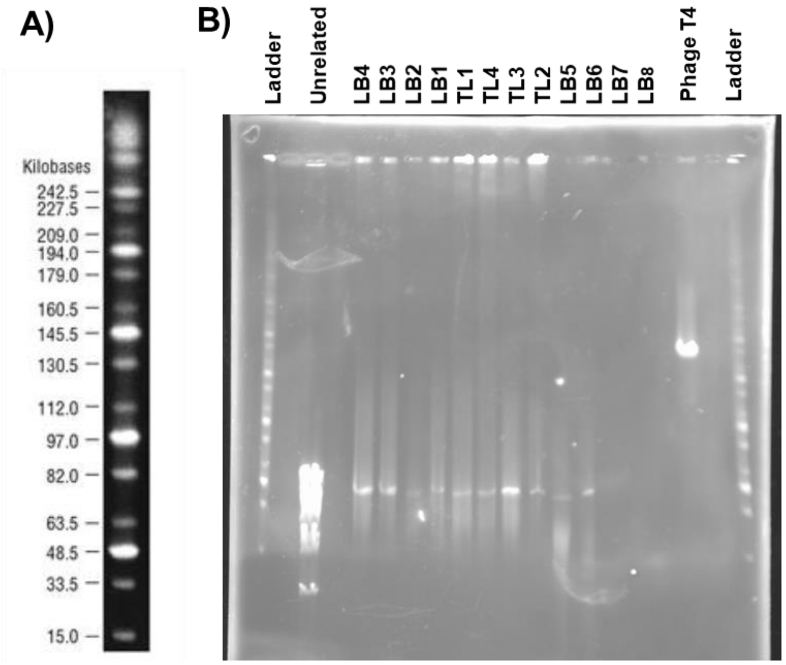

C)

Phage ID	Phage Concentration Pfu/mL
LB8	2.05 X 10^{4}
LB7	2.23 X 10^{5}
LB6	5.30 X 10^{9}
LB5	3.70 X 10^{9}
TL2	2.45 X 10^{9}
TL3	2.45 X 10^{9}
TL4	4.30 X 10^{9}
TL1	2.35 X 10^{8}
LB1	1.00 X 10^{12}
LB2	2.25 X 10^{9}
LB3	2.25 X 10^{10}
LB4	2.25 X 10^{10}

Supplemental Figure S1. Determination of phage genome sizes by PFGE. A) Molecular size ladder B) Pulse Filed Gel Electrophoresis. C) Phages concentration used for Pulse Field Gel Electrophoresis plugs. Lysate names are used in the figure. Each lysate contains the following phage genomes: LB1 = maay; LB2 = tuull; LB3 = hasilly; LB4 = kwarr; LB5 = shaaii and ehaak; LB6 = nyashin; LB7 = emuu; LB8 = enyaa, nyashin, and CF418-P1; TL1 = nyaak; TL2 = kuwaak; TL3 ; TL4 = ewik. Lysates LB7 and LB8 did not contain enough phage DNA to be visualized.

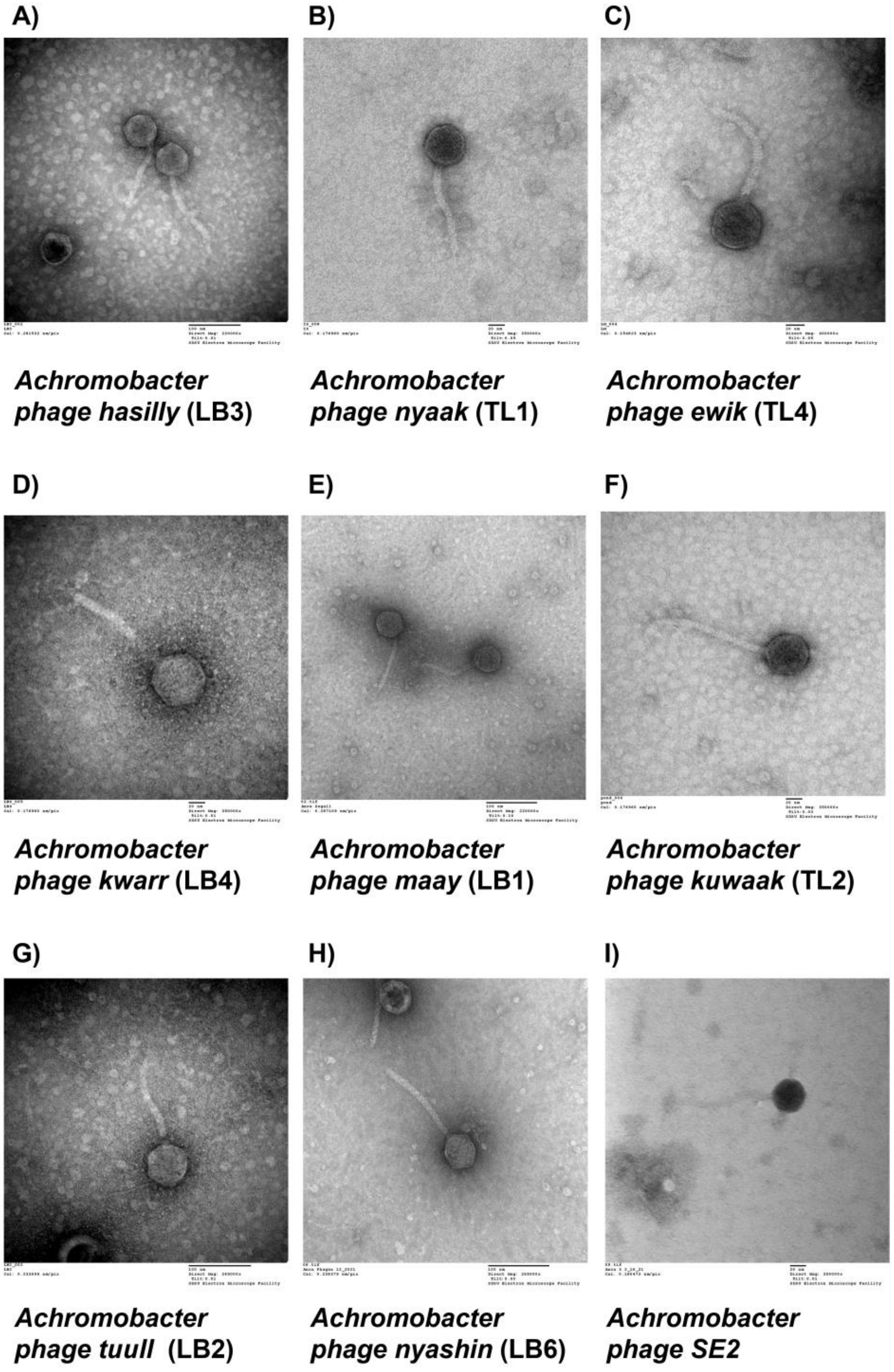

Supplemental Figure S2. *Achromobacter* phages transmission electron microscopy. Images for pure phage lysates are shown. *Achromobacter* phages *shaii*, *ehaak*, *emuu* and *enyaa* were in mixed lysates and images are not shown.

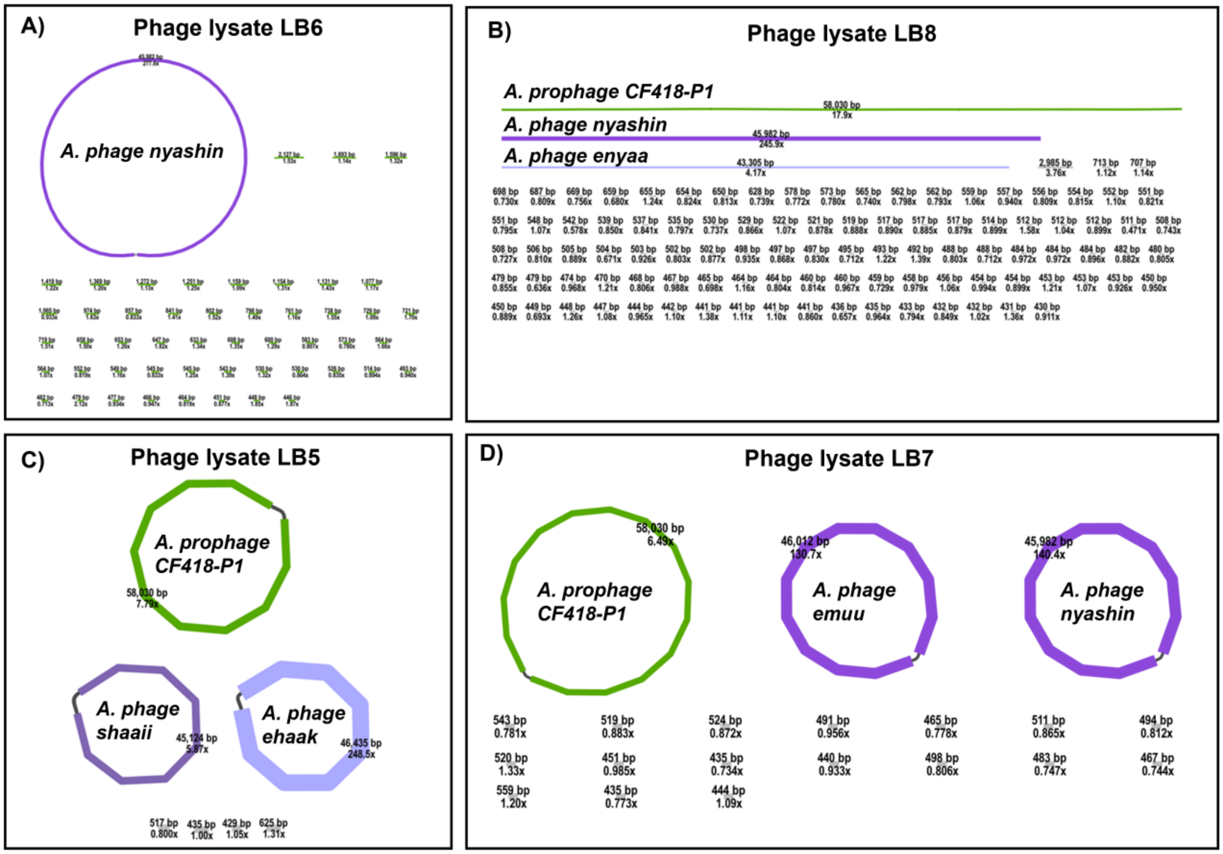

Supplemental Figure S3. Prophage induced in *Achromobacter* CF418 when infected with additional lytic phages. Contigs assembled from each phage lysate using SPADES were visualized in BANDAGE. A) Assembly with 100,000 reads. One bacteriophage genome present. B) Assembly with 100,000 reads. Two bacteriophage genomes present, one with high coverage of 250X (light purple) and another one with low coverage 5X (dark purple). One *Achromobacter* element present (green) C) Assembly with 100,000 reads. Two bacteriophage genomes present, one with high coverage of 250X (light purple) and another one with low coverage 5X (dark purple). One *Achromobacter* element present (green) D) Assembly with 100,000 reads. Two bacteriophage genomes present with similar coverage (dark purple). Achromobacter element present (green).

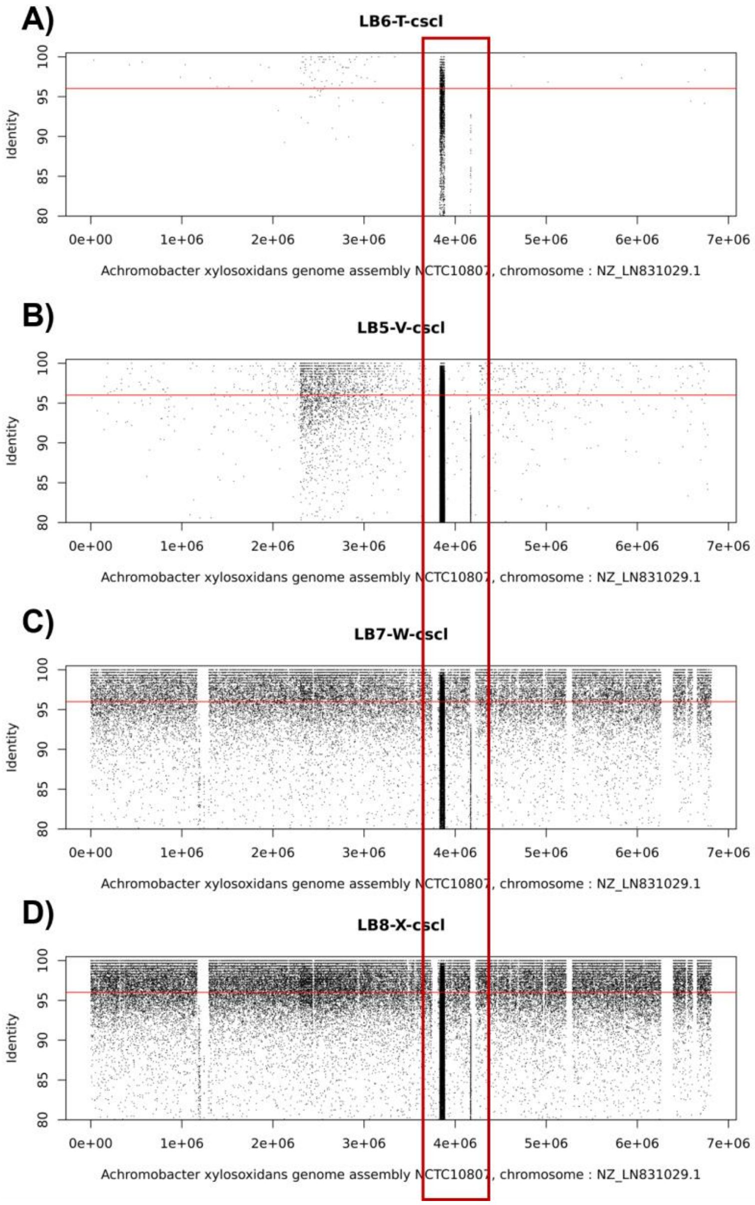

Supplemental Figure S4. Prophage induced in *Achromobacter* CF418 when infected with lytic phages. Genome coverage of bacterial reference genome. Region in the red box is the prophage CF418-P1.

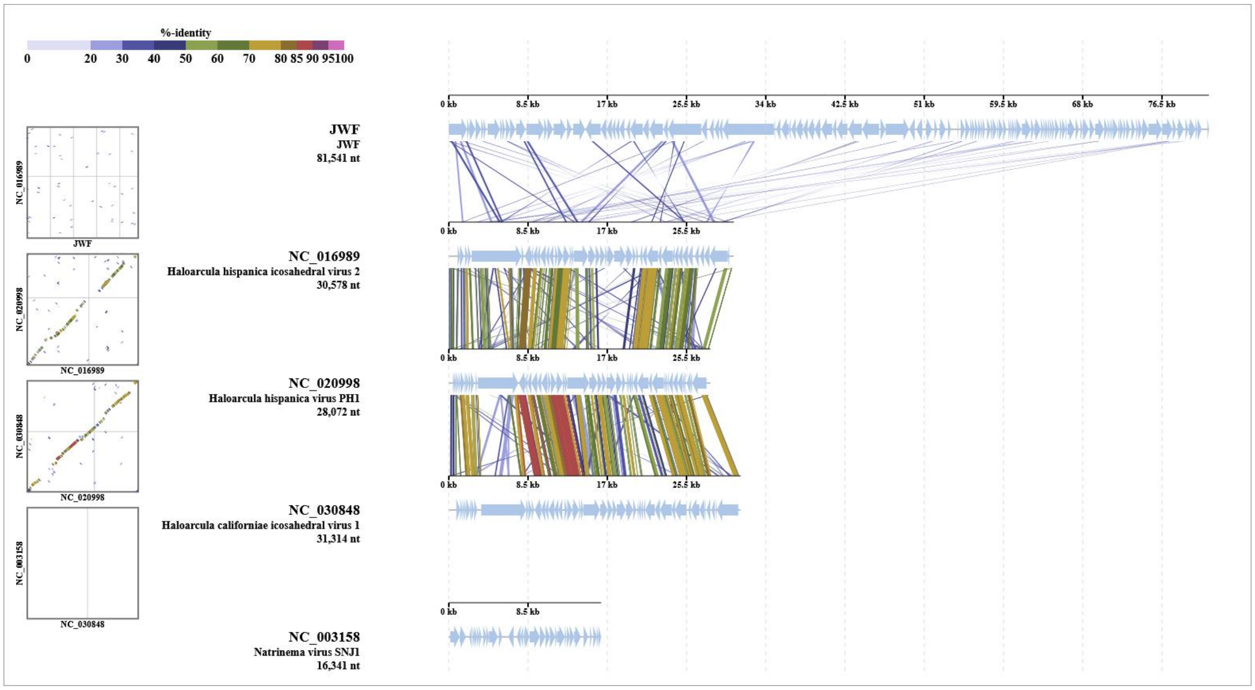

Supplemental Figure S5. Phage Proteomic Tree alignment visualization of *Achromobacter* phage JWF and its closest relatives.

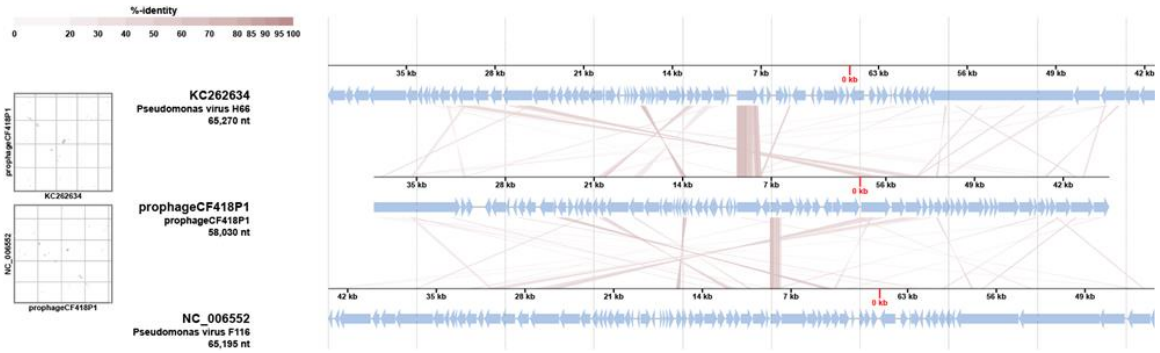

Supplemental Figure S6. Genome comparison of Achromobacter prophage CF418-P1 and its closest phage relatives.

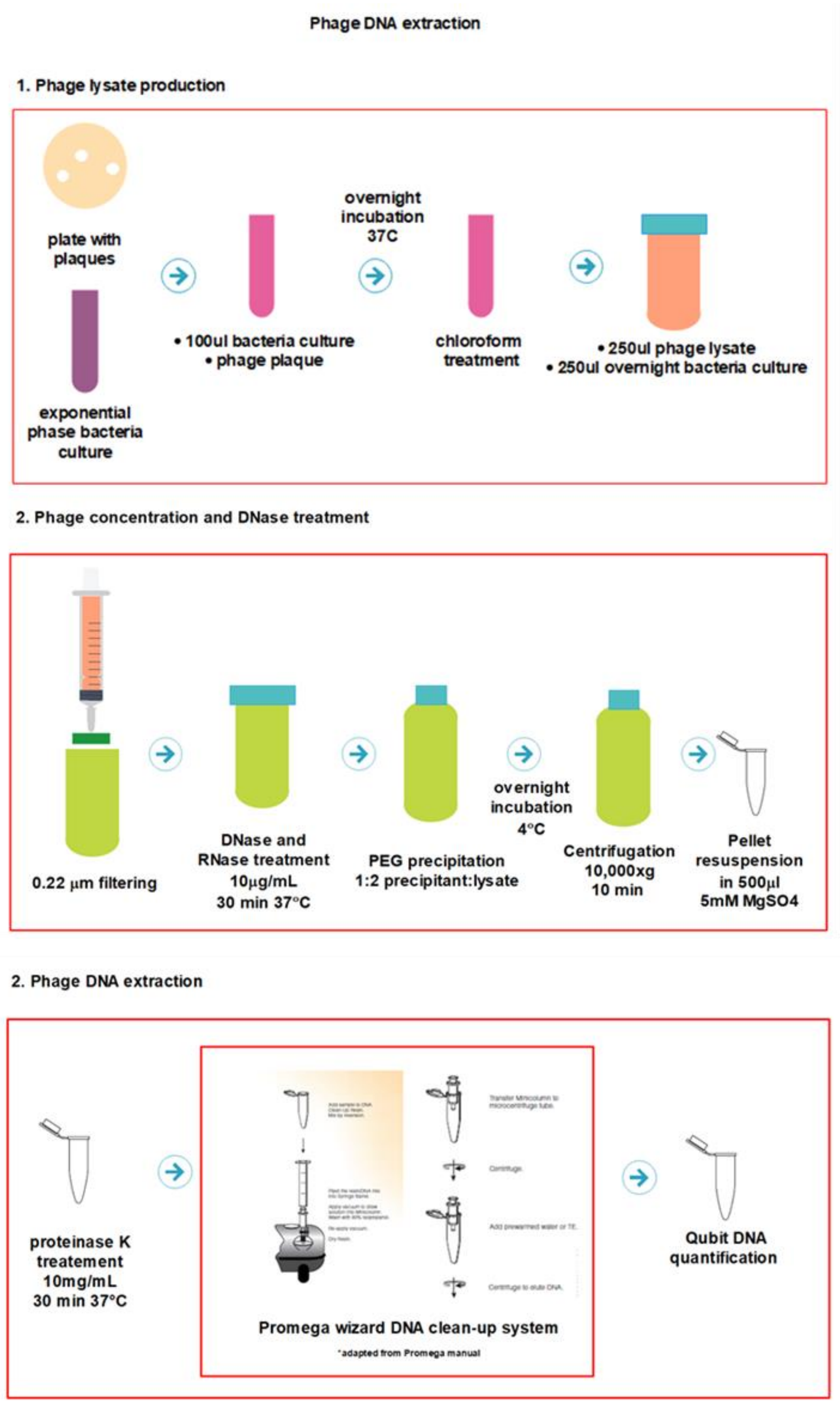

Supplemental Figure S7. Protocol for phage propagation and DNA extraction for genome sequencing.

Supplemental Table S1. *Achromobacter* lytic bacteriophage genomes in the literature (n=24)

Phage name	Genome length (bp)	Accession number	Clade	Morphology (virus family)	Reference	Propagation strain
Achromobacter phage vB_AXyS_19-32_Axy04	73,834	MK962626	JWAlpha	Podoviridae	(Essoh C. et al, 2020)	*Achromobacter xylosoxidans* I2BC
Achromobacter phage vB_AXyS_19-32_Axy06	45,830	MK962627	JWX	Siphoviridae	(Essoh C. et al, 2020)	*Achromobacter xylosoxidans* I2BC
Achromobacter phage vB_AXyS_19-32_Axy09	43,287	MK962628	Axy09	Podoviridae	(Essoh C. et al, 2020)	*Achromobacter xylosoxidans* I2BC
Achromobacter phage vB_AXyS_19-32_Axy10	73,898	MK962629	JWAlpha	Podoviridae	(Essoh C. et al, 2020)	*Achromobacter xylosoxidans* I2BC
Achromobacter phage vB_AXyS_19-32_Axy11	73,413	MK962630	JWAlpha	Podoviridae	(Essoh C. et al, 2020)	*Achromobacter xylosoxidans* I2BC
Achromobacter phage vB_AXyS_19-32_Axy12	74,096	MK962631	JWAlpha	Podoviridae	(Essoh C. et al, 2020)	*Achromobacter xylosoxidans* I2BC
Achromobacter phage vB_AXyS_19-32_Axy13	70,103	MK962632	JWAlpha	Podoviridae	(Essoh C. et al, 2020)	*Achromobacter xylosoxidans* I2BC
Achromobacter phage vB_AXyS_19-32_Axy14	46,703	MK962633	JWX	Siphoviridae	(Essoh C. et al, 2020)	*Achromobacter xylosoxidans* I2BC
Achromobacter phage vB_AXyS_19-32_Axy16	46,178	MK962634	JWX	Siphoviridae	(Essoh C. et al, 2020)	*Achromobacter xylosoxidans* I2BC
Achromobacter phage vB_AXyS_19-32_Axy18	45,500	MK962635	phiAxp1	Siphoviridae	(Essoh C. et al, 2020)	*Achromobacter xylosoxidans* I2BC
Achromobacter phage vB_AXyS_19-32_Axy19	46,036	MK962636	phiAxp1	Siphoviridae	(Essoh C. et al, 2020)	*Achromobacter xylosoxidans* I2BC
Achromobacter phage vB_AXyS_19-32_Axy20	46,352	MK962637	phiAxp1	Siphoviridae	(Essoh C. et al, 2020)	*Achromobacter xylosoxidans* I2BC
Achromobacter phage vB_AXyS_19-32_Axy21	43,049	MK962638	Axy09	Podoviridae	(Essoh C. et al, 2020)	*Achromobacter xylosoxidans* I2BC
Achromobacter phage vB_AXyS_19-32_Axy22	71,710	MK962639	JWAlpha	Podoviridae	(Essoh C. et al, 2020)	*Achromobacter xylosoxidans* I2BC
Achromobacter phage vB_AxyS_19-32_Axy23	43,773	MK962640	Axy09	Podoviridae	(Essoh C. et al, 2020)	*Achromobacter xylosoxidans* I2BC
Achromobacter phage vB_AxyS_19-32_Axy24	74,744	MK962641	JWAlpha	Podoviridae	(Essoh C. et al, 2020)	*Achromobacter xylosoxidans* I2BC
Achromobacter phage phiAxp-1	45,045	NC_029033.1	phiAxp1	Siphoviridae	(Li E, et al., 2016)	*Achromobacter xylosoxidans* A22732
Achromobacter phage phiAxp-2	62,220	NC_029106.1	phiAxp2	Siphoviridae	(Li E, et al., 2016)	*Achromobacter xylosoxidans* A22732
Achromobacter phage phiAxp-3	72,825	NC_028908.2	JWAlpha	Podoviridae	(Ma Y, et al., 2016)	*Achromobacter xylosoxidans* A22732

Achromobacter phage 83-24	48,216	NC_028834.1	JWX	Siphoviridae	(Rohde M, et al., 2017)	*Achromobacter xylosoxidans* HER 83-190
Achromobacter phage JWX	49,714	NC_028768.1	JWX	Siphoviridae	(Rohde M, et al., 2017)	*Achromobacter xylosoxidans* LMG 3465
Achromobacter phage JWF	81,541	NC_029075.1	JWF	Siphoviridae	(Rohde M, et al., 2017)	*Achromobacter xylosoxidans* CCUG 48136
Achromobacter phage JWDelta	73,659	KF787094.1	JWAlpha	Podoviridae	(Wittmann J, et al., 2014)	*Achromobacter xylosoxidans* DSM 11852
Achromobacter phage JWAlpha	72,329	NC_023556.1	JWAlpha	Podoviridae	(Wittmann J, et al., 2014)	*Achromobacter xylosoxidans* DSM 11852

Supplemental Table S2. Phage lysates and phage genomes names

Isolation ID	Sequencing ID	Phage contig ID	Phage name	Host	Phage isolation source
IS	TL1	*Achromobacter phage nyaak* TL1	*Achromobacter phage nyaak*	*A. ruhlandii* CF116	Influent water sample
pond	TL2	*Achromobacter phage kuwaak* TL2	*Achromobacter phage kuwaak*	*A. ruhlandii* CF116	SDSU fishpond water
LM	TL4	*Achromobacter phage ewik* TL4	*Achromobacter phage ewik*	*A. ruhlandii* CF116	Lake Murray
IS2	LB2	Achromobacter phage *tuull* LB2	Achromobacter phage *tuull*	*A. ruhlandii* CF116	Influent water, site 2. San Elijo Joint Powers Authority. 2695 Manchester Avenue, Cardiff by the Sea, CA, 92007-7077.
IS1	LB1	Achromobacter phage maay LB1	Achromobacter phage maay	*A. ruhlandii* CF116	Influent water, site 1. Water Quality Laboratory. 1521 S. Hale Avenue, Escondico, CA, 92029.
IS3	LB3	Achromobacter phage hasilly LB3	Achromobacter phage hasilly	*A. ruhlandii* CF116	Influent water, site 3. Encina Wastewater Authority. 6200 Avenida Encinas, Carlsbad, CA, 92011.
SA2	LB5	Achromobacter phage LB5-A	Achromobacter phage ehaak	*A.ruhlandii* CF418	Infuent water
SA3	LB5	Achromobacter phage LB5-B	Achromobacter phage shaaii	*A.ruhlandii* CF418	Infuent water
S315S	LB7	Achromobacter phage LB7-A	Achromobacter phage emuu	*A.ruhlandii* CF418	Influent water
S313L	LB8	Achromobacter phage LB8-B	Achromobacter phage enyaa	*A.ruhlandii* CF418	Influent water
S2D	LB6-repeated	Achromobacter phage nyashin LB6 (repeated in 3 samples)	Achromobacter phage nyashin	*A.ruhlandii* CF418	Influent water
S313L	LB8-prophage	Achromobacter prophage CF418-P1 (prophage from CF418)	Achromobacter prophage CF418-P1	*A.ruhlandii* CF418	Influent water
IS4	LB4	Achromobacter phage LB4 (partial genome ?)	Achromobacter phage kwarr	*A. ruhlandii* CF116	Influent water, site 4. Vallecitos Water District. 7941 Corintia Street, Carlsbad, CA.

Supplemental Table S3. Illumina sequencing information for Achromophages

Phage lysate	Illumina good quality reads	*Hits to A. ruhlandii* NZ_CP017433.1	*Hits to A. xylosoxidans* NZ_LN831029.1	% *Achromobacter* reads	Hits to human genome	% Human genome reads
TL2-B-filtered	1,894,772	4	1	0.0003	3	0.0000
TL3-C-filtered	1,749,597	3	2	0.0003	0	0.0000
LB1-D-filtered	2,537,881	12	6	0.0007	1	0.0000
LB2-E-filtered	705	0	4	0.5674	0	0.0000
LB3-F-filtered	2,274,283	14	12	0.0011	0	0.0000
LB4-G-filtered	406	0	0	0.0000	0	0.0000
TL1-M-cscl	3,406,503	73	12	0.0025	10	0.0001
TL4-U-cscl	5,142,519	315	105	0.0082	35	0.0001
LB5-V-cscl	4,040,869	207	112,219	2.7822	0	0.0000
LB6-T-cscl	1,874,759	4	3,970	0.2120	0	0.0000
LB7-W-cscl	3,895,260	1,826	102,389	2.6754	145	0.0002
LB8-X-cscl	2,473,235	3,115	183,814	7.5581	23	0.0001

Supplemental Table S4. Achromophages Nanopore sequencing information.

Sample type	Sample ID	DNA amount (nanograms)	BARCODE	Reads	% total reads
Phage	TL1	397.50	RB01	772	0.84
Phage	TL2	400.00	RB02	1867	2.04
Phage	TL3	400.00	RB03	1642	1.79
Phage	TL4	203.25	RB04	2700	2.95
Phage	LB4	60.75	RB05	5023	5.49
Phage	LB3	18.00	RB06	1127	1.23
Phage	LB7	10.50	RB09	585	0.64
Phage	LB8	3.26	RB10	231	0.25
Bacteria	Achromo CF418	213.75	RB12	21470	23.47
			Unclassified	35399	38.70
			Total	91482	

Supplemental Table S5. Lifestyle prediction for Achromobacter phages.

**Phage name**	**Lifestyle**	**probability of temperate lifestyle**	**sd for temperate lifestyle**	**probability of lytic lifestyle**	**sd for lytic lifestyle**	**Integrase in genome**
Achromobacter phage nyaak		0.514	0.041	0.486	0.041	0
Achromobacter phage kuwaak		0.512	0.040	0.488	0.040	0
Achromobacter phage ewik		0.509	0.045	0.491	0.045	0
Achromobacter phage *tuull*		0.504	0.043	0.496	0.043	0
Achromobacter phage maay		0.517	0.042	0.483	0.042	0
Achromobacter phage hasilly		0.509	0.041	0.491	0.041	0
Achromobacter phage ehaak		0.486	0.043	0.514	0.043	0
Achromobacter phage shaaii	Lytic	0.468	0.039	**0.532**	**0.039**	0
Achromobacter phage emuu		0.482	0.039	0.518	0.039	0
Achromobacter phage enyaa		0.505	0.043	0.495	0.043	0
Achromobacter phage nyashin	Lytic	0.461	0.039	**0.539**	**0.039**	0
Achromobacter prophage CF418-P1	Temperate	**0.526**	**0.040**	0.474	0.040	**1**
Achromobacter phage kwarr		0.508	0.037	0.492	0.037	0

Supplemental Table S6. Predicted prophages in *Achromobacter xylosoxidans* CF418. Predictions were made using PHASTER.

Prophage region	Length	Completeness	Score	Number of proteins	Region position	Most common phage	GC%
1	31Kb	intact	130	20	2394771-2425793	PHAGE_Burkho_Bcep176_NC_007497(7)	66.43%
2	26.2Kb	questionable	90	31	4871986-4898219	PHAGE_Pseudo_F116_NC_006552(4)	66.58%
3	17.5Kb	incomplete	60	20	4966675-4984247	PHAGE_Salmon_SEN34_NC_028699(13)	66.65%
4	19.4Kb	incomplete	50	12	5270924-5290399	PHAGE_Caulob_CcrPW_NC_048046(2)	65.66%

Supplemental Table S7. Prophage CF418-P1 genome annotation.

Feature ID	Type	Start position	End position	Strand	Protein length	Predicted function
fig|1416009.21.repeat.1	repeat	1	127	+		
fig|1416009.21.peg.1	CDS	1831	32	-	600	Terminase
fig|1416009.21.peg.2	CDS	1853	1969	+	39	
fig|1416009.21.peg.3	CDS	1985	2140	+	52	Minor tail
fig|1416009.21.peg.4	CDS	2221	2913	+	231	
fig|1416009.21.peg.5	CDS	2972	3070	+	33	
fig|1416009.21.rna.1	rna	3102	3019	-	28	tRNA-Val-AAC
fig|1416009.21.peg.6	CDS	3820	3134	-	229	
fig|1416009.21.peg.7	CDS	4412	3807	-	202	
fig|1416009.21.peg.8	CDS	5077	4409	-	223	Endodeoxyribonuclease RusA
fig|1416009.21.peg.9	CDS	6288	5137	-	384	Tail fiber
fig|1416009.21.peg.10	CDS	7118	6216	-	301	DNA methyltransferase
fig|1416009.21.peg.11	CDS	7783	7067	-	239	
fig|1416009.21.peg.12	CDS	9780	7783	-	666	C-5 cytosine-specific DNA methylase
fig|1416009.21.peg.13	CDS	9957	11510	+	518	
fig|1416009.21.peg.14	CDS	11429	11950	+	174	
fig|1416009.21.peg.15	CDS	12015	12437	+	141	
fig|1416009.21.peg.16	CDS	12455	13072	+	206	Lipoprotein
fig|1416009.21.peg.17	CDS	13201	13554	+	118	
fig|1416009.21.peg.18	CDS	13551	13658	+	36	
fig|1416009.21.peg.19	CDS	13655	14152	+	166	
fig|1416009.21.peg.20	CDS	14149	14358	+	70	
fig|1416009.21.peg.21	CDS	14355	14639	+	95	
fig|1416009.21.peg.22	CDS	14636	14824	+	63	
fig|1416009.21.peg.23	CDS	14838	15641	+	268	
fig|1416009.21.peg.24	CDS	15657	15878	+	74	
fig|1416009.21.peg.25	CDS	15881	17083	+	401	Recombinase RecT
fig|1416009.21.peg.26	CDS	17099	18259	+	387	DNA translocase FtsK
fig|1416009.21.peg.27	CDS	18269	20053	+	595	
fig|1416009.21.peg.28	CDS	20069	20683	+	205	DNA Polymerase III
fig|1416009.21.peg.29	CDS	20686	21174	+	163	
fig|1416009.21.peg.30	CDS	21171	21815	+	215	
fig|1416009.21.peg.31	CDS	21815	22288	+	158	
fig|1416009.21.peg.32	CDS	22291	22572	+	94	
fig|1416009.21.peg.33	CDS	22576	23889	+	438	
fig|1416009.21.peg.34	CDS	23949	24056	+	36	
fig|1416009.21.peg.35	CDS	24150	25298	+	383	Integrase
fig|1416009.21.peg.36	CDS	25381	25581	+	67	

fig|1416009.21.peg.37	CDS	25615	26385	+	257	
fig|1416009.21.peg.38	CDS	26473	27000	+	176	SOS response-associated protein
fig|1416009.21.peg.39	CDS	27106	27405	+	100	
fig|1416009.21.peg.40	CDS	27441	27554	+	38	
fig|1416009.21.peg.41	CDS	27554	27661	+	36	
fig|1416009.21.peg.42	CDS	27692	27979	+	96	
fig|1416009.21.peg.43	CDS	28000	29115	+	372	Antitoxin HigA
fig|1416009.21.peg.44	CDS	29134	29619	+	162	
fig|1416009.21.peg.45	CDS	29968	29747	-	74	
fig|1416009.21.peg.46	CDS	30149	30003	-	49	
fig|1416009.21.peg.47	CDS	30409	30212	-	66	Myosin tail containing protein
fig|1416009.21.peg.48	CDS	31075	30530	-	182	Endopeptidase
fig|1416009.21.peg.49	CDS	31482	31072	-	137	Peptidoglycan hydrolase
fig|1416009.21.peg.50	CDS	39602	31548	-	2685	
fig|1416009.21.peg.51	CDS	41393	39612	-	594	Dehydrogenase E1
fig|1416009.21.peg.52	CDS	42580	41393	-	396	
fig|1416009.21.peg.53	CDS	43055	42582	-	158	
fig|1416009.21.peg.54	CDS	43339	43052	-	96	
fig|1416009.21.peg.55	CDS	44005	43361	-	215	
fig|1416009.21.peg.56	CDS	44296	44006	-	97	
fig|1416009.21.peg.57	CDS	45465	44296	-	390	Minor tail
fig|1416009.21.peg.58	CDS	47123	45468	-	552	Minor tail
fig|1416009.21.peg.59	CDS	47434	47126	-	103	
fig|1416009.21.peg.60	CDS	47786	47436	-	117	
fig|1416009.21.peg.61	CDS	48226	47819	-	136	
fig|1416009.21.peg.62	CDS	49482	48226	-	419	Collar, Peptidase
fig|1416009.21.peg.63	CDS	49947	49486	-	154	
fig|1416009.21.peg.64	CDS	50493	49960	-	178	
fig|1416009.21.peg.65	CDS	50884	50495	-	130	Head tail junction, surface antigen repeat
fig|1416009.21.peg.66	CDS	51529	50888	-	214	
fig|1416009.21.peg.67	CDS	52176	51526	-	217	
fig|1416009.21.peg.68	CDS	52601	52176	-	142	Peptidase
fig|1416009.21.peg.69	CDS	53105	52686	-	140	Minor tail
fig|1416009.21.peg.70	CDS	54456	53158	-	433	
fig|1416009.21.peg.71	CDS	55536	54478	-	353	
fig|1416009.21.peg.72	CDS	57938	55656	-	761	
fig|1416009.21.repeat.2	repeat	57904	58030	+		
fig|1416009.21.peg.73	CDS	58030	57935	-	32	DNA packaging

Supplemental Table S8. Isolated Achromophages for broader host range.

Phage ID	Phage name	Phage isolation host	Phage isolation source	Phage isolation source collection date
M1	*Achromobacter phage M1*	*Achromobacter* sp. VVP0357	Vallecitos Wastewater District. (Meadowlark Water Reclamation Facility), Carlsbad, CA.	2/20/2019
M2	*Achromobacter phage M2*	*Achromobacter* sp. VVP0357	Vallecitos Wastewater District. (Meadowlark Water Reclamation Facility), Carlsbad, CA.	2/20/2019
ENA1	*Achromobacter phage ENA1*	*Achromobacter* sp. VVP0357	Encina Wastewater Authority, Carlsbad CA. 92011	2/20/2019
MW2	*Achromobacter phage MW2*	*Achromobacter* sp. VVP0426	Vallecitos Wastewater District. (Meadowlark Water Reclamation Facility), Carlsbad, CA.	2/20/2019
SE2	*Achromobacter phage SE2*	*Achromobacter* sp. VVP0426	San Elijo Joint Powers Authority, Cardiff by the Sea, CA.	4/16/2019
